# Ultrasonication: An Efficient Alternative for the Physical Modification of Starches, Flours and Grains

**DOI:** 10.3390/foods13152325

**Published:** 2024-07-24

**Authors:** Antonio J. Vela, Marina Villanueva, Felicidad Ronda

**Affiliations:** 1 Department of Agriculture and Forestry Engineering, Food Technology, College of Agricultural and Forestry Engineering, University of Valladolid, 34004 Palencia, Spain; antoniojose.vela@uva.es (A.J.V.); marina.villanueva@uva.es (M.V.); 2 Whistler Center for Carbohydrate Research, Department of Food Science, Purdue University, West Lafayette, IN 47907, USA; 3 Research Institute on Bioeconomy-BioEcoUVa, PROCEREALtech Group, University of Valladolid, 47011 Valladolid, Spain

**Keywords:** ultrasonic treatment, structural and morphological properties, pasting properties, rheological properties, starch digestibility

## Abstract

Ultrasonic (USC) treatments have been applied to starches, flours and grains to modify their physicochemical properties and improve their industrial applicability. The extent of the modification caused by USC treatment depends on the treatment conditions and the natural characteristics of the treated matter. Cavitation leads to structural damage and fragmentation and partial depolymerization of starch components. The amorphous regions are more susceptible to being disrupted by ultrasonication, while the crystalline regions require extended USC exposure to be affected. The increased surface area in USC-treated samples has a higher interaction with water, resulting in modification of the swelling power, solubility, apparent viscosity, pasting properties and gel rheological and textural properties. Starch digestibility has been reported to be modified by ultrasonication to different extents depending on the power applied. The most important treatment variables leading to more pronounced modifications in USC treatments are the botanical origin of the treated matter, USC power, time, concentration and temperature. The interaction between these factors also has a significant impact on the damage caused by the treatment. The molecular rearrangement and destruction of starch structures occur simultaneously during the USC treatment and the final properties of the modified matrix will depend on the array of treatment parameters. This review summarizes the known effects of ultrasonic treatments in modifying starches, flours and grains.

## 1. Introduction

Starch is the energy reserve polysaccharide of various plants and one of the most abundant carbohydrates in nature. It is found in many different plant organs including seeds, fruits, tubers and roots [1]. Starches are found in the form of partially crystalline structures insoluble in water, having different size, morphology, composition, molecular weight and physicochemical properties depending on their botanical origin [2,3]. Starch granules mainly consist of a mixture of two polysaccharides: amylose and amylopectin. Amylose is essentially a linear macromolecule consisting of a α-D-glucan chain linked through α-(1→4) linkages with a degree of polymerization (DP) between 1000 and 10,000 glucose units [4,5]. Only a small portion of amylose (0.1%) are branched via α-(1→6) linkages. Amylopectin, on the other hand, is a highly branched macromolecule composed mainly of α-(1→4) linked D-glucopyranose (as in amylose) linked through nonrandom α-(1→6) linkages at a greater proportion than amylose [4,5]. Both components differ in their molecular weight, degree of ramification and chemical properties. Starches show concentric rings of alternating layers of amorphous and crystalline structures. The amorphous regions consist of amylose and amylopectin chains in a disordered conformation, while the crystalline rings are formed by the double helices in clusters of amylopectin branches [6]. The ratio and proportion of these components generally range from 20 to 25% for amylose and from 75 to 80% for amylopectin, depending on the source of starch [7].

Starches are the main source of carbohydrates in human diet, supplying almost two thirds of the required daily calories [2] and representing a valuable ingredient in a wide variety of applications in the food industry (i.e., thickening, gelling agent, bulking agent, water retention agent and adhesive) [4,8]. Starch is the main component in flours and grains, with other primary nutrients like proteins, fat and other carbohydrates [9]. Starch intake in the human diet derives from the consumption of food products containing flours as an ingredient and cooked grains (e.g., rice, maize, oat, quinoa, buckwheat), a basic food in different cultures all over the world [10].

However, despite its several industrial uses, the proprieties of native starches do not usually fulfill the industry’s specific requirements because of limitations such as low shear resistance, thermal resistance and a high tendency toward retrogradation [11]. As a solution to this matter, native starch granules can be modified through genetic, mechanical, chemical, enzymatic or physical modifications to obtain improved properties [8,12]. In recent years there has been an increasing attraction toward physical modifications, especially in food applications, leading to similar properties to those obtained in chemically modified starches but without the need to use chemical agents, hence being cleaner and more environmentally friendly procedures. Some physical modification methods of starches and flours include extrusion, hydrothermal treatments, microwave radiation and ultrasound [8]. The advantages of physical modification methods are that they require short processing time, have higher selectivity and are classified as “green technology”, resulting in increasing research interest observed in recent years. Furthermore, physically modified starches/flours are better accepted by consumers, particularly in food applications, given that they do not require any chemical or biological agent.

The aim of this review paper is to summarize the research that has been published regarding the modification of starches, flours and grains by ultrasonic treatments and to explain the results that have been published, comparing the reasons for contradictory results among different studies.

## 2. Physical Modification by Ultrasonic Treatments

Among the physical modification methods of starches and flours, ultrasonic (USC) treatments have shown many advantages in terms of higher selectivity, efficiency and quality, requiring a shorter processing time, representing reduced physical and chemical risks and reducing waste generation and energy consumption [12,13,14,15]. The term ultrasound refers to mechanical waves with a frequency above 18 kHz that originate from either a piezoelectric or magnetostrictive transducer within high frequency electrical fields that create high-energy vibrations [1,13]. These vibrations are later amplified and transferred to a probe/sonotrode or a bath in direct contact with the fluid to be treated [7]. Ultrasound waves need an elastic medium to spread over, so treatments are always performed in suspensions [14]. Water is the most commonly used solvent in these modifications, given that it is the safest solvent for food applications. USC can be divided into three frequency regions: low-frequency ultrasound (also known as power ultrasound) in the region of 16 to 100 kHz, high-frequency ultrasound in the range of 100 kHz to 1 MHz and diagnostic ultrasound from 1 to 10 MHz [7].

**Figure 1:**
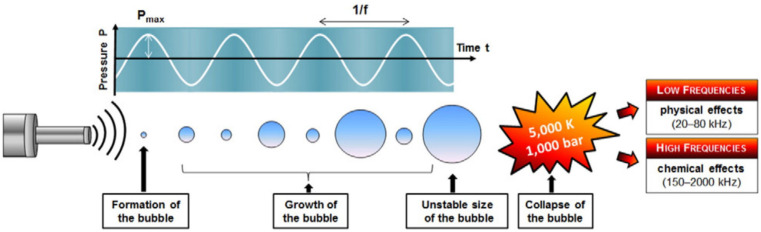
Schematic representation of the acoustic cavitation phenomenon [17].

In USC treatments, the acoustic energy cannot be directly absorbed by the molecules of the medium but it is transformed to a usable form by the cavitation phenomenon. The sinusoidal ultrasound waves pass through the aqueous medium, inducing a longitudinal displacement of particles. This results in a rapid and successive cyclical movement of compression and rarefaction phases in the medium, creating multiple tiny collapsing bubbles, which constitute the cavitation phenomenon (see Figure 1) [14,16,17,18]. When bubbles collapse, high energy is released and converted to high pressure (up to 20 MPa) and hot spots (high temperatures up to 5000 °C) that can generate both physical and chemical effects in the modification of starches and flours [10,13]. The physical effects of cavitation include intense microjets streaming with high velocity (hundreds of m/s) toward the surface of the treated particles in a very short time, shear forces and shock waves generated by the collapsing bubbles [19]. During collapse, the bubble becomes asymmetric and its wall accelerates more on the side opposite to the solid surface, resulting in the formation of a strong microjet of water directed toward the particle’s surface [20], which brings material fatigue followed by a gradual tearing off of microscopic particles capable of breaking polymeric chains [16]. Furthermore, the turbulent flow generated by sonication can also induce granule–granule collision and the collision between the starch granules and the wall of the container where they are hosted during treatment, which can result in starch granule damage [20]. On the other hand, cavitation can also have a chemical effect through the generation of free radicals, such as hydroxide (-OH) and hydrogen (-H), as a result of the dissociation of the solvent molecules [3,19], which may lead to a chemical modification of the system [12,14]. It has been generally observed that low-frequency ultrasounds lead to a greater mechanical effect over the granules while high-frequency ultrasounds are widely used in food applications where the generation of free radicals is needed [15,17].

The total amount of energy released by cavitation depends on the kinetics of the bubble growth and collapse. The size of bubbles varies depending on the applied frequency [3]. The theoretical resonance size at 20 kHz is around 150 µm, while at a frequency of 1 MHz, the resonance size is considered to be about 3 µm [2]. Hence, it is estimated that low-frequency ultrasounds produce relatively large bubbles compared to the starch granular size (5–80 µm), releasing more energy and inducing greater shear damage on the granule surface after collapsing [2]. Also, at lower frequencies, there is more time to form the bubbles, which increases the energy of the shock waves [21]. The solvent used for sonication also influences the effect of cavitation over the treated particles. Cavitation intensity increases with surface tension at the bubble interface and decreases with increasing vapor pressure of the medium; thus, liquids having small surface tension require lower energy to produce bubbles, resulting in cavitation occurring more readily [22]. However, the use of different solvents with different surface tensions is not common in the modification of starches/flours using USC; almost all the literature available works with water.

**Figure 2:**
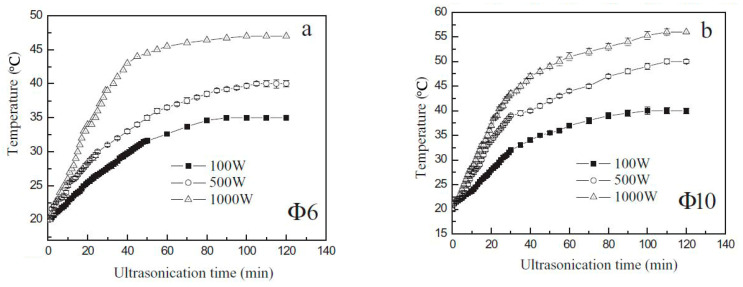
Relationship of ultrasonication time and temperature of rice starch suspension under different ultrasound powers and intensities under treatments using a probe with a titanium tip of (**a**) 6 mm and (**b**) 10 mm diameter [23].

Cavitation can significantly increase the temperature of the starch suspension, having a strong thermal effect on the modified matter. Yu et al. (2013) determined that higher temperatures are reached when sonicating at higher power (see Figure 2) [23]. Since USC treatments are always performed in aqueous dispersions, the annealing process always occurs simultaneously as a side effect of ultrasonication. Annealing refers to a physical modification method of starches and flours performed in excess water (>60%) at a temperature below the onset temperature of gelatinization [24]. If the temperature increase is not controlled and surpasses the onset temperature of starch gelatinization, the starch will swell and gelatinize during treatment, making this a critical treatment variable [13,25]. Temperature affects the vapor pressure of water in which elevated temperatures decrease the transmitted energy, resulting in reduced cavitation intensity [13].

Ultrasonic treatments can affect the starch/flour dispersions in at least three ways: (a) causing a physical degradation of the particles; (b) leading to a reduction in the molecular weight of amylose and amylopectin as a result of the breakage of C-C bonds; and (c) solubilizing swollen starch granules, including “ghost” granules that remain even after the complete gelatinization of the starch dispersion [15]. These effects depend strongly on the treatment conditions, such as applied frequency, power, amplitude, time and temperature as well as the conditions of the treated suspension, such as the biological origin of the starch/flour, solvent used, suspension concentration and treated amount [12,26]. It was determined by Cui and Zhu (2020) that the extent of structural and physicochemical changes induced by ultrasonication not only depends on the type of plant material but also on varietal differences, after obtaining significant differences between the modification of purple dawn and red sweet potato flours using the exact same treatment conditions [10]. The variety of the sample defines the structure of the starch, and the structure controls the physicochemical properties of the sample and its vulnerability to modification by USC. 

A comparison of the published experimental results regarding the modification of starches, flours and grains by USC is not straightforward, since the effects are strongly dependent on complex experimental conditions arrays. Several studies have investigated the properties of ultrasound-modified starches, flours and grains from different botanical origins and confirmed different effects on the morphological, molecular, physicochemical, functional, rheological and digestion properties depending on the applied treatment conditions (see Table 1) (refer to Table S1 for a detailed summary). It is the objective of this review to summarize and organize the results obtained so far in the literature, to explain how USC treatments depend on the operating conditions and the matrices treated and to understand the feasibility of this technology to obtain ingredients with improved properties. The most important findings are presented below.

## 3. Effect of Ultrasound Modifications on Morphological Properties

### 3.1. Surface Damage

It has been extensively indicated that ultrasounds lead to surface damage, which in starch granules has been described as cracks [13,20,21,27,28,29,30], holes [31,32], pits [2,16,33,34,35,36], pores [32,37,38,39,40], scratches [19,41,42], roughness [43,44], grooves [45,46], fissures [26,47,48,49], fractures [50] and channels [51,52], in agreement with the damage reported in flour particles [10,25,53,54] and even rice grains [55]. This damage derives from the cavitation phenomenon, attributed to high pressure causing shear forces on starch granules surfaces [26,31], which might even induce the destruction of starch granules [35]. Some studies did not report any effect of ultrasonication on granules surface, which might be due to low sonication power during treatment or the application of USC by a bath, given that more marked effects have been reported when sonicating with a probe [7,15].

**Figure 3:**
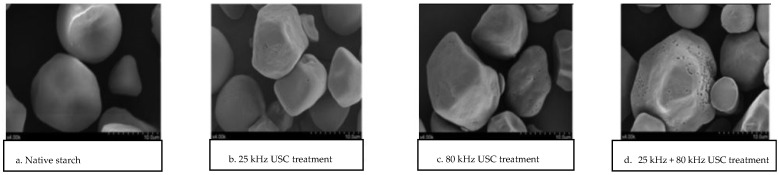
SEM photos of sweet potato starch samples sonicated for 60 min at different frequencies (4000×). Untreated starch (**a**), ultrasonicated at 25 kHz (**b**), ultrasonicated at 80 kHz (**c**), double USC treatment at 25 kHz and 80 kHz (**d**) [8].

The porosity of the granules influences the chemical reactivity of starches, as pores, channels and cavities increase the starch surface area and allow reagents and enzymes to penetrate more easily into the bulk of the granule, potentially accelerating chemical and enzymatic reactions [3,8,38]. It is generally accepted that lower frequencies, longer times and higher temperatures lead to more noticeable surface damage. It is highly dependent on the frequency applied given that it limits the size at which bubbles collapse. The number of pits per granule would increase with increasing sonication frequencies because they result in much smaller bubbles [2]. Furthermore, Zheng et al. (2013) determined a higher number of dents and holes in sweet potato starch after treatment by dual frequency (25 + 80 kHz) than what was observed in single frequency treatments (25 or 80 kHz) [8] (see Figure 3). Dual-frequency ultrasound, meaning two beams of USC propagating together at the same time in the treated solution, could cause greater damage to starches because the cavitation yield is higher and the bubbles collapse faster than in single-frequency treatments [8,56].

**Figure 4:**
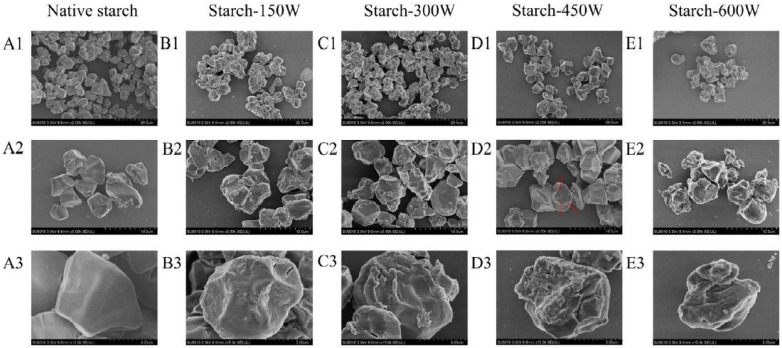
Scanning electron micrographs of native rice starch (**A1**–**A3**) and rice starch treated by 150 W (**B1**–**B3**), 300 W (**C1**–**C3**), 450 W (**D1**–**D3**) and 600 W (**E1**–**E3**) ultrasound. The magnification of image from top to bottom in the same line was 2, 5 and 15 K, respectively [40].

The damage caused by USC to the starch surface is aggravated at higher ultrasonic power [20,34,46,57]. Yang, Kong et al. (2019) determined that the outer layer of rice starch granules was gradually peeled off from the periphery as the power increased from 150 W to 600 W [40] (see Figure 4). In agreement with these findings, Ding et al. (2019) and Wang, Lv et al. (2022) demonstrated that roughness and erosion of starch granules appeared in a power-dependent manner in treatments from 100 W to 600 W and from 200 W to 600 W, respectively [43,58]. Longer sonication [2,21,30,59] and higher temperatures [25,32,41,44,60] have widely been indicated to intensify the surface damage induced by ultrasonication. Flores-Silva et al. (2017) determined that while 4 min showed fissures and cracks, 8 min led to severe disruption of the granule surface and 16 min caused granule fragmentation and disintegration [37]. In agreement, Bel Haaj et al. (2013) indicated that the surface of waxy maize starch granules appeared to be progressively broken down and eroded with increasing sonication time, releasing nanoparticles of about 20–200 nm [6]. The treatment temperature can also lead to surface damage [15], being able to cause a partial gelatinization of the surface [44] and even disintegrate some starch granules [13,32,41] and flour particles [25]. It has been said that even when the temperature of the treated suspension is controlled during treatment, USC can lead to local heating that damages the outer regions of the granules, increasing the disruptive effects induced by cavitation [61].

The susceptibility of starches to ultrasonication is also related to their botanical origin. It has been said that the degree of damage caused to granules depends on their size, given that the relative distance between granules and cavitation bubbles is a factor determining the transition from a microjet mechanism to a shear force during cavitation [20]. Hu et al. (2019) demonstrated that USC caused greater damage to larger granules (potato) than to small granules (millet), in agreement with Carmona-García et al. (2016), since larger granules have a greater probability of trapping kinetic energy [32,61]. Susceptibility is also related to the type and structure of the starch, including the amylose/amylopectin ratio [12,26]. Luo et al. (2008) noted that under the same treatment conditions, normal maize and waxy maize starches showed a porous surface after ultrasonication, while a fissure was clearly observed in the surface of amylomaized V starch [26]. Furthermore, Chan et al. (2010) determined that when sonicating different types of starches (sago, potato, corn and mung bean) under the same conditions, fissures were only found on corn starch granules, possibly attributed to a relatively weaker granular structure [48].

The nature and degree of erosion caused by cavitation also depend on the concentration of starch in the suspension [13,21], the solvent used [3] and the gas present in the medium during treatment [62]. The damage has been said to be greater at lower concentrations because of increased acoustic energy due to the reduced impedance of the medium [13], while at higher concentrations the density of the acoustic energy is reduced due to diffusion of the waves by the particle [21]. Regarding the medium, cavities are more readily formed in solvents with high vapor pressure, low viscosity and low surface tension [3]. Water has a high surface tension and low viscosity and vapor pressure, making it a good medium for cavitation and degradation of starch [3]. The solubility of the surrounding gas in water has been found to be inversely proportional to the size of pits generated in starch granules [21]. It was determined by Gallant et al. (1972) that in the presence of air, the surface of potato starch granules became rugged and pitted, whereas in an atmosphere of hydrogen, the surface remained smooth with large and deep pits. In the presence of oxygen and carbon dioxide, there was less detectable damage, and virtually no effect was produced in a vacuum [21].

### 3.2. Particle Size

The available literature indicates that the effect of USC on particle size depends greatly on treatment factors. Some authors have reported the rupture of starch granules and flour particles by the collapse of cavitation bubbles, reducing the size of particles [5,25,32,40,45], while others indicated slight changes [15,50,51], no effects at all [31,34,42,61] or even a granule size increase [30,33,35,43,63] after ultrasonication.

**Figure 5:**
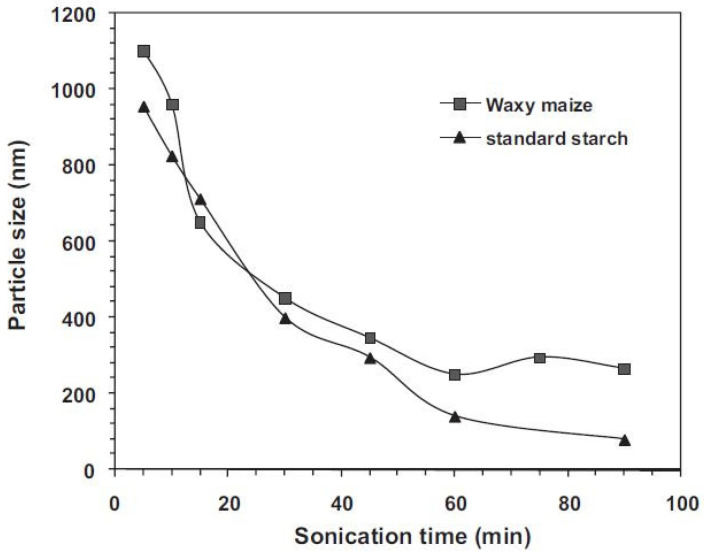
Change in mean particle size vs. ultrasonication time for waxy maize and normal maize starch (only particles larger than 100 nm were taken into consideration) [6].

The device used for applying ultrasound waves seems to greatly determine the effect on particle size. Fewer effects have been reported when using an ultrasound bath [1,57] but the size of granules was greatly reduced when using probes [7]. When treating with a USC bath, the granules tend to agglomerate due to superficial adhesiveness among granules and the liberated bonds, providing the opportunity of connecting linkages between the polymers, which results in increased sizes after ultrasonication [1,9,33]. When sonicating with a probe, the generation of small-sized particles has been commonly reported. Degrois et al. (1974) and Gallant et al. (1972) were the first authors to report the physical degradation of starch granules after ultrasonic treatments [21,62]. When a significant size reduction is determined, it is believed that the progressive erosion caused by mechanical collision and shear forces from cavitation leads to granule fragmentation [6,40]. Bel Haaj et al. (2013) said that particle reduction was mainly caused by the violent collision of starch particles due to high-speed streams resulting from the implosion of the bubbles rather than the effect of the direct impact of the microjets on the particles [6]. The concentration of the suspension, the temperature and time of ultrasonication and the nature of the treated sample (e.g., amylose content) also influence the extent of fragmentation achieved [45,64]. Greater particle fragmentation has been reported in starches and flours with increasing sonication time [10] until a limiting size is reached (see Figure 5) [6,53]. Minakawa et al. (2019) determined that amylose content positively influenced particle reduction, obtaining smaller starch microparticles (1–3 μm) and nanoparticles (8–32 nm) after ultrasonic treatment of yam starch (higher amylose) than corn and cassava starches [64].

### 3.3. Color

Color in starches and flours is an important quality parameter determining their acceptability and applications. The extent of color change induced by ultrasonication differs among samples depending on their nature and the treatment conditions. In starches, not much research has been carried out on color given their lack of pigmentation, which leaves room for fewer changes [18,28]. In pigmented flours, different authors have said that L* could be either increased or decreased by USC, which seemed to depend on sonication time. Vela, Villanueva, Ozturk, et al. (2023) reported an increase in L* in tef flours at a short ultrasonication time (10 min), originating mainly from particle size reduction, which increased surface area and allowed more light reflection [24]. However, in much longer treatments, 20 h for Cui and Zhu (2020) and 19.2 h for Zhu and Li (2019), lower L* values were reported (it is worth mentioning that in these studies, USC was not applied the entire time but annealing did cause a modification during the whole treatment time) [10,65]. It is believed that long soaking times could lead to the darkening of flours due to the action of oxidase enzymes, which could be enhanced by mechanical or thermal treatments [10]. The water removal method can also influence the color evolution due to the loss of soluble pigments [66].

## 4. Effect of Ultrasounds on Structural Properties

### 4.1. Molecular Structure of Starch—Molecular Weight and Chain Length Distribution

Many of the physical and functional properties of starches depend on their molecular conformation, like molecular weight, chain length distributions and amylose to amylopectin ratio, which can be modified by ultrasonication [14]. Depolymerization as a consequence of ultrasonication can involve two mechanisms: (i) mechanical polymeric degradation due to cavitation and (ii) chemical degradation resulting from reactions between the polymer and -OH radicals and -H atoms generated by the dissociation of water molecules due to cavitation [7,14]. Free radicals may induce the scission of starch molecular chains, which disrupts the fine molecular structure and thus destroys the integrity and rigidity of starch granules [30]. Polymeric degradation through chemical reactions stands out at high-frequency ranges (>500 kHz) while in low-frequency treatments, the main effect is due to mechanical degradation [3].

Starch depolymerization has been extensively reported after ultrasonic treatments [7,8,13,16,28]. The experimental conditions determine the yield of polymer degradation caused by ultrasound, in which severe conditions promote the breakage of starch macromolecules (longer time, higher temperature, higher power). Temperature has been reported to have a direct correlation with the diminishment of the degree of polymerization (DP) while an inverse correlation has been reported for starch concentration in the suspension [13,60]. The molecular weight of starches has been indicated to be reduced by ultrasonication [4,19,45,67,68], where the reduction has been reported to be sharp at the beginning of treatment, to slow down as treatment progresses and to ultimately tend toward a constant value [69]. A characteristic feature of polymer degradation through USC is that it proceeds in a nonrandom manner and that there is a minimum chain length limiting the degradation process [16]. It has been demonstrated that the shear forces of cavitation do not have a significant impact on small molecules but are capable of breaking polymeric chains since they are more vulnerable as longer chains with more bonds are susceptible to being impacted [16]. Once chains become shorter, their vulnerability to fragmentation is reduced.

**Figure 6:**
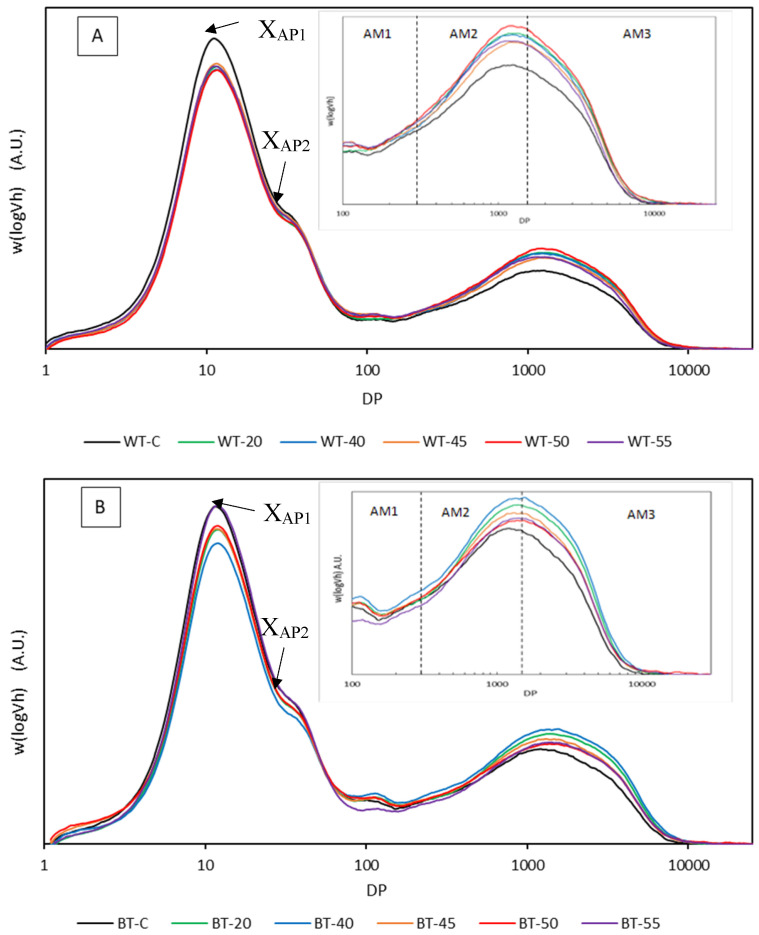
Size exclusion chromatograms of debranched white (**A**) and brown (**B**) starch samples with enlargement of the amylose regions as function of DP [54].

The amylose to amylopectin ratio in starches differs depending on the nature of each starch. It is believed that ultrasonic treatments preferentially degrade (but not exclusively) linear amylose of the amorphous regions with low structural integrity [13,26,51], since linear polymeric conformations may accumulate the applied forces of the same spatial orientation on much longer distances along the chain [16] while the destruction of crystalline regions and the unwinding of double helices requires more energy [38,40,45]. The distortion of the crystalline regions has been reported after highly intense ultrasonication (>420 W) of corn [19] and rice [40] starches. Starch depolymerization by high power occurs mainly on the C-O-C bond of the α-(1→6)-glycosidic bonds (the branching points of amylopectin), resulting in amylopectin gradually being converted to low molecular weight segments [19,31]. The degradation of the side chains and partial depolymerization of amylopectin molecules would also increase the apparent amylose content (AAC) given that it enlarges the number of linear fragments and reduces the steric hindrance effect of long internal chains, which allows iodine to bind with them [19,43,67,70]. Furthermore, USC might affect the mobility of the molecules in the amorphous and crystalline regions, leading to the organization and formation of amylose–amylose, amylose–amylopectin and amylopectin–amylopectin helical structures that increase AAC [58]. Figure 6 presents how size-exclusion chromatographs of white and brown tef starches were modified by USC treatments, resulting in increased relative areas corresponding to the polymerization degree range of amylose chains (degree of polymerization [DP] > 100) [54]. Higher amylose contents have been reported with increasing sonication time [36,70] and USC power [34,58,69,71].

### 4.2. Changes in the Crystalline and Chemical Structure of Starch

#### 4.2.1. Polarized Light Microscopy (PLM)

Polarized light microscopy has been used to observe the crystal structure of starch granules. The density and refractive index difference between crystalline and amorphous structures generates the Maltese cross, clearly seen when granules are exposed to polarized light [19,46]. These Maltese crosses are characteristic of intact granules and reflect their crystalline organization [36,38]. Ultrasonication has either been reported to cause no change or to decrease the luminance of the Maltese cross. When no effect has been found, it has been concluded that USC cannot change the whole granular structure but may change parts of it (likely the amorphous parts), barely affecting the crystalline structure [38,46]. On the contrary, whenever a brightness decrease (or damage) is detected in the Maltese cross, it has been attributed to disruption of the crystal layer of granules by ultrasonication, reducing the order of the molecular chain and leaving a more fragile structure [19,21,35,61].

#### 4.2.2. X-ray Diffraction (XRD)

XRD allows the evaluation of X-ray patterns and degree of long-range order crystallinity in starches. Based on XRD data, native starches can be classified into types “A”, “B” or “C”. There seems to be a general consensus in the literature indicating that USC treatments do not modify the position of the characteristic peaks, while the diffraction intensities of the sonicated starches have been reported to remain unchanged [25,31,35,39,46,52,72] or to be reduced [13,19,27,43,44,45,50,63,73]. The susceptibility of starches to ultrasonication is influenced by the packing of their crystalline and amorphous regions [32,51]. It has been indicated that the characteristic peak intensities experienced a greater reduction at longer USC exposure [30,50,61] and higher temperatures [13].

In starches, XRD patterns, peak diffraction characteristics and dispersion diffraction characteristics correspond to the crystalline and the amorphous regions, respectively [74]. The long-range order crystallinity of the starch, also called relative crystallinity (RC), can be determined as the ratio between the crystalline and total region [47]. In agreement with the lower XRD pattern intensities reported after USC treatments, RC has been commonly indicated to be reduced by ultrasonication in A-type (rice [34,40], corn [19,44,74], waxy corn [45], normal maize [6,35], oat [31], tapioca [59], millet [32], quinoa [70], canary seed [73] and sweet potato [36,49]), B-type (potato [46] and retrograded starch RS3 [43]) and C-type (pea [60,67], cassava [52] and pinhão [75]) starches. Lower RC values have been attributed to damage caused to the amorphous regions rather than the crystalline regions because of their higher susceptibility to ultrasonication [40,76]. This may result from the breakage of hydrogen bonds and starch chain structures by cavitation and mechanical oscillation pressure during USC treatments, causing the destruction of amorphous regions, the loose packing of lattices and the transformations of double-helix orientation, leading to a decrease in RC [36]. However, the inner lamellae (presumably more crystalline and richer in amylopectin chains) are also susceptible to the attack of ultrasonic waves [37,52]. It is believed that intensified USC conditions (both power and time) [31,40,46] reduce RC because they can disrupt the double-helix structure of the crystalline regions [35,36]. Bel Haaj et al. (2013) determined that prolonged ultrasonication of starch under strong treatment conditions resulted in serious disruption of the crystalline structure, leading to nanoparticles with low crystallinity [6]. Small crystallites may present an amorphous character and could result in a lower RC [59]. Even when ultrasonication can damage the starch crystalline structure, their breakdown strength is usually not enough to induce a change in the diffraction pattern [74].

**Figure 7:**
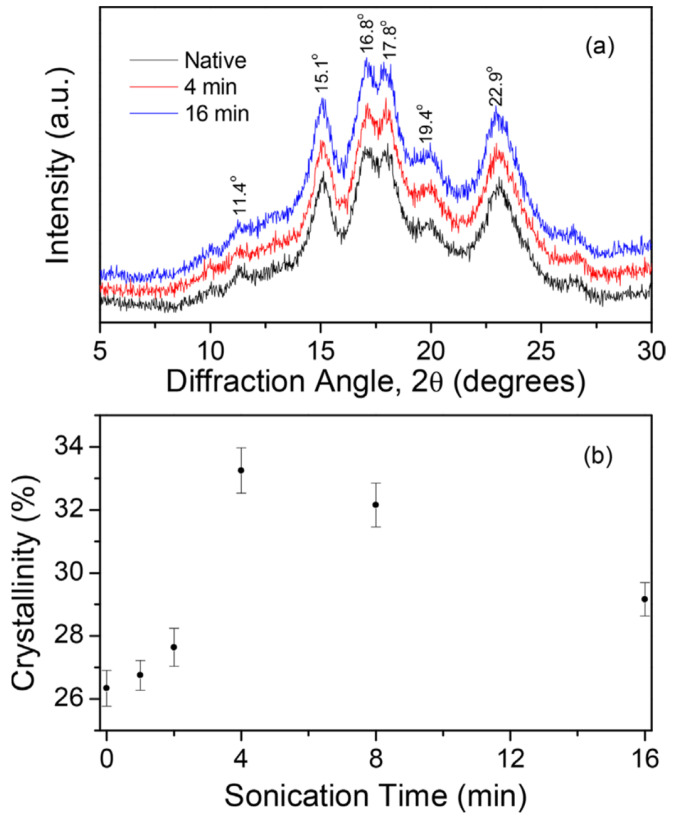
(**a**) XDR pattern for native starch and two sonicated samples; (**b**) crystallinity content determined for different treatment times [37].

Some studies have reported no RC change after ultrasonication, while a few authors even reported an increase following treatments. These results tend to be attributed to preferential degradation of the amylose-rich amorphous regions by ultrasonication, leaving the crystalline structure rather unaffected [26,29,38,57,77]. It may be assumed that in these studies, the treatment conditions were less prone to lead to an effect over the crystalline regions rather than a lower susceptibility of the starch structure to ultrasonication [51]. Flores-Silva et al. (2017) reported an RC increase from about 25% in the native corn starch to about 33% after 4 min sonication (see Figure 7) [37]. These authors concluded that USC did not lead to a modification in the composition of the starch molecules but only their relative organization within the granule microstructure. Cleavage of starch chains in the amorphous regions allows the formation of new crystallites and some reordering of the fragmented chains that produce a more crystalline structure [33,72]. USC performed at high temperatures has been said to reduce RC in starches (pea [60] and corn, pea and potato [41]), while increased values have been reported in flours (rice [25,55] and tef [54]). These results suggest that flours might be more tolerant to high-temperature USC. The disruption of crystalline structure and rearrangement of starch molecules occur simultaneously during USC and it seems like A-type starches might be more prone to using the thermal energy to moderate molecular rearrangements. Ouyang et al. (2021) concluded that RC in corn (A-type diffraction pattern) showed increased values at low temperatures (5–25 °C) but reduced values at higher temperatures (35–45 °C), while the B- and C-type starches showed decreased values at all temperatures. It is believed that the low amount of water within the A-type starch structure allows the molecule to assemble into a more ordered structure [41]. Furthermore, these results suggest that A-type flours might be more tolerant to high-temperature USC than A-type starches given that RC in corn starch was reduced at 35–45 °C while rice and tef flours still showed increased RC values at 60 and 55 °C, respectively.

#### 4.2.3. Fourier Transform Infrared Spectroscopy (FTIR)

FTIR is used to verify changes in the chemical structures of starch molecules resulting from USC treatments [5]. The bands associated with vibrations, stretching, flexion and deformation of bonds corresponding to the main functional group characteristics of starch are in the region of 800–1200 cm^−1^ [44,51]. Ultrasonic treatments have been indicated to modify the shape, width and intensity of FTIR spectra but the positions of the characteristic absorption peaks are not significantly changed [49,50,52]. Neither the loss of absorption peaks nor the generation of new ones has been reported after treatments, suggesting that USC does not alter chemical bonds and functional groups, indicative of a purely physical modification [2,19,27,32].

The absorbance bands at 1047 cm^−1^, 1022 cm^−1^ and 995 cm^−1^ are particularly sensitive to modifications caused to starches and are associated with the ordered (crystalline) structure, the amorphous structures and bonding in hydrated carbohydrate helices, respectively [37,52]. The 1047/1022 ratio is assumed to reflect the amount of ordered structure in starch [77]. It has been generally reported that USC treatments reduce the 1047/1022 values, indicative of disruption of short-range molecular order [28]. It is presumed that this decrease occurs because ultrasonication destroys the amorphous and crystalline regions of starch granules, resulting in irregular packing with a double-helical reorientation within crystalline domains and/or the disruption of some hydrogen bonds linking adjacent double helices [19,30,49]. This ratio has presented a decreasing tendency with increasing USC power, suggesting a weakening of starch short-range crystallinity [34,40]. Sonication time, on the other hand, does not seem to have a direct correlation with the decrease in 1047/1022 values. Flores-Silva et al. (2017) determined a significant decrease in the 1047/1022 ratio in USC treatments from 1 min to 4 min (when the lowest value was reached), which remained constant until 16 min sonication, indicating that the limit of short-range crystallinity crackdown is achieved even in short treatments [37]. Similarly, Vela, Villanueva, Solaesa, et al. (2021) found no significant differences in this ratio in rice flour sonicated for different times (2–60 min) [53]. On the contrary, Wang, Xu et al. (2020) concluded that time influenced the decrease in 1047/1022 values [30]. These different results are believed to arise from other factors influencing the effect of treatments, like the botanical origin of the sample (i.e., amylose content, amylose/amylopectin ratio and size of the granules) in synergy with the treatment conditions (power, concentration, time, etc.). Zhang et al. (2021) demonstrated that different starches presented different trends in the evolution of 1047/1022 values after being ultrasonicated under the same conditions. While sonicated pea and potato starches showed a remarkable decrease in these values, sonicated corn starch presented much higher values than its control, with a constant increase with increasing time [71]. Whenever a 1047/1022 increase has been reported, it has been explained as more serious damage caused to the amorphous regions and determined that ultrasounds enhanced the associations between starch chains and favored the formation of relatively ordered structure (single- and double-helices) due to the recrystallization of short chains [39,43,71]. Another evaluated ratio, although to a much lower degree, is 1022/995, assumed to represent the organization state of the double helices located inside the crystallites [52]. Usually, a decrease in 1047/1022 is joined by an increase in 1022/995, indicative of a higher proportion of amorphous to ordered structure zones in the sonicated starches [44,52], confirming the general weakening of short-range order [32,40].

FTIR spectroscopy has also been used to evaluate the effect of USC on proteins when treatments were applied to flours. Changes in the protein secondary structure are evaluated in the range of 1700–1600 cm^−1^, corresponding to amide I, the most prominent vibrational band of the protein backbone structure [53]. Changes in this band have been estimated from the relative area of each individual peak in the deconvoluted amide I curve, assigned to α-helix, β-sheet, β-turn and random coil. The available literature indicates that α helix and β-sheet structures are particularly reduced by USC treatments, while β-turn and random coil are increased [25,53,54,66]. The random coil increase suggests a rearrangement of polymeric subunits that leads to an increase in the disordered structure [54]. These differences after ultrasonication derive from the action of shear forces, disrupting the interactions between protein molecules and influencing the protein molecule internal structure [53]. However, there is not much literature regarding this subject and deeper study is needed to reach solid conclusions.

#### 4.2.4. Raman Spectroscopy

The starch short-range ordered structure has also been studied by Raman spectroscopy, which has been reported to be more sensitive than FTIR to local changes in polymer microstructure [6]. Vibrations related to the C-O-C of α-1,4 and α-1,6 glycosidic linkages in starches are characterized by strong bands at 900–960 cm^−1^ and a weak band at 1155 cm^−1^. A significant modification of these bands was reported by Bel Haaj et al. (2013), where the band at 905 cm^−1^ seemed to vanish, the band at 940 cm^−1^ was shifted toward a lower wavenumber and the band at 1155 cm^−1^ decreased in intensity. Based on these results, the authors concluded that the branching points in amylopectin were mostly affected by high-power ultrasonication since the band at 905 cm^−1^ is associated with α-1,6 glycosidic linkages [6]. The 480 cm^−1^ Raman band has been reported to have a strong correlation with the short-range order structure of starch and has been used to characterize changes due to USC treatments. Wang, Xu et al. (2020) used the full width at half height at 480 cm^−1^ to characterize this crystallinity value, while Yang, Kong et al. (2019) used the height of the band for this determination [30,40]. A lower degree of short-range molecular structure was suggested by both studies, reaching the conclusion that USC weakens the ordered packing of double-helix structures in starch granules. This conclusion agrees with the commonly reduced values of the 1047/1022 ratio reported by FTIR analyses (see Section 4.2.3). Furthermore, the intensity of the band at 2900 cm^−1^ has been reported to be reduced as ultrasonication intensifies, which might be related to distortion of the ordered molecular structure, closely associated with changes in the amylose/amylopectin ratio [30,40].

#### 4.2.5. Nuclear Magnetic Resonance Spectroscopy (NMR)

**Figure 8:**
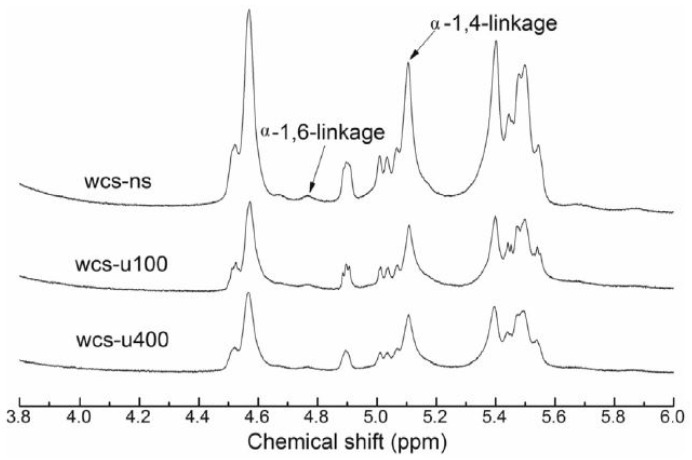
^1^H NMR spectra of native and ultrasonicated waxy corn starches. wcs-ns, wcs-u100 and wcs-u400 refer to the waxy corn starch that was not sonicated, sonicated at 100 W and sonicated at 400 W, respectively [45].

To confirm structural changes in starch due to sonication, some authors have recorded the proton and carbon nuclear magnetic resonance spectroscopy (^1^H NMR and ^13^C NMR, respectively). The high sensitivity of ^1^H NMR allows the resolution of the anomeric proton resonance of starch, distinguished between the α-1,4 and α-1,6 glycosidic linkages, to determine the degree of branching (DB) of native and ultrasonicated starches [45,72]. Signals at 5.12 ppm and 4.80 ppm are assigned to α-1,4 and α-1,6 glycosidic bonds, respectively [72]. After ultrasonication, Acevedo et al. (2022) and Hu et al. (2023) reported a decrease in the 4.80 ppm signal, while Yang, Lu et al. (2019) and Vela, Villanueva, Li, et al. (2023) indicated a relative decrease in both peaks (see Figure 8) [45,54,63,72]. Consequently, the DB in the sonicated starches was decreased, associated with a decrease in the anomeric signal α-(1,6) [45,63,72]. These results agree with the higher amylose content reported as result of the destruction of amylopectin branches. Iida et al. (2008) reported that the integrated intensity of the peaks increased with sonication, indicative that the fraction of the mobile starch molecules increased due to the treatment [4]. In the case of waxy starches, the steric hindrance of α-1,4 glycosidic linkages was more stable than that of α-1,6 glycosidic linkages, hence being more resistant to ultrasonication [45,78]. These results suggest that ultrasounds break both α-1,4 and α-1,6 glycosidic linkages, with preferential rupture of one or the other depending on the USC power applied and the amylose content in the starch.

In ^13^C NMR spectra, each carbon of the glucose unit appears as a single line and six main peaks are attributed to C-1 (90–110 ppm), C-4 (80–84 ppm), C-6 (58–65 ppm) and the overlapping signal of C-2, C-3 and C-5 (68–79 ppm) [4,45]. The two broad shoulder peaks near 103 and 82 ppm provide information about the amorphous components in starch while the triplet peaks in the C-1 region provide information about the crystalline state [45]. It was determined by Yang, Lu et al. (2019) that as the ultrasound power increased, the signals representing the amorphous state were gradually intensified while the intensity of the peaks in the crystalline state was reduced [45]. Iida et al. (2008) found that ^13^C NMR spectral intensity was largely increased by sonication, supporting the argument that the highly mobile fraction of starch increased with ultrasonication [4]. Both results indicate that the crystalline state decreased and the amorphous state increased with USC. The increase in the single helix might be because the crystalline region was destroyed and double helices were unwound into many short single helices and amorphous states [45].

## 5. Effect of Ultrasounds on Techno-Functional Properties

Techno-functional properties of starches and flours are ultimately related to the interaction they have with water, which deeply depends on the starch granule morphology and composition [48]. The sonication process provides heat to the medium during treatment that can result in a temperature increase and as it has been indicated that temperature influences to a great extent the modification caused by ultrasonication; so, it is necessary to use a cooling system to control the temperature of the medium during treatment. It was indicated by Monroy et al. (2018) that the complete starch sample gelatinized during USC treatment when an ice bath was not used, given that the slurry reached 65 °C under 20 min sonication [52].

### 5.1. Swelling Power and Solubility

Swelling power (SP) and solubility (S) are parameters closely related to the starch granule fine structure, gelatinization temperature, amylose to amylopectin ratio, degree of branching and branch length, conformation and molecular weight degree of association between their chains and the aggregation structure of the granules [30,42,48,51]. These properties are linked together because when the granule swells, more amylose can be released to the aqueous medium, so high swelling power contributes to high solubility [48].

An increase in SP and S has been commonly reported after USC treatments of starches [18,31,48,50,77], flours [25,53] and grains [76]. As a consequence of starch surface damage after ultrasonication, water diffuses more easily into the granules, so USC-treated samples present higher water uptake and retention, increasing their SP [7,26]. The generation of nanoparticles also favors starch interaction with water since small-sized granule fragments have a higher specific surface area than large native granules [31]. The increase in SP and S can also be a consequence of starch depolymerization and the disassociation of ordered molecular structure. Higher SP and S indicate improved binding between water molecules and free hydroxyl groups of starch polymer chains through hydrogen bridges, suggesting that the molecular disruption by ultrasounds contributed to the increase in linear chains [26,30,42,59]. Ultrasonication enhanced amylose chain mobility and reduced their molecular weight after the cleavage of amylose chains, thus improving their hydration performance and promoting the leaching of amylose outside the swollen granules to the aqueous medium [1,48]. Within the crystalline structures, the disintegration of intermolecular bonds and the release of side chains lead to a less compact granular arrangement, generating free hydroxyl groups of amylose and amylopectin where water molecules can bind with hydrogen bonds, also increasing SP [51].

The final effect of USC over these properties depends on the treatment parameter array, where time and temperature are highly influential. Short sonication times (below 30 min) have been said to increase the SP of starches [18,50,52] while longer times lead to lower values [39]. This behavior is believed to happen because, with the increase in starch damage under a long exposure, there is a reduction in the starch structural stability, which detriments its SP [39]. Solubility, on the contrary, has been reported to be increased by longer exposure, favored by the disruption of the amorphous regions [8,59]. It was indicated by Karwasra et al. (2020) that S increased in wheat starches at short times (15 min) but decreased under sonication for 30 min [50]. This tendency switch is believed to be related to the temperature reached during ultrasonication since these authors did not report to have controlled the treatment temperature, which would have resulted in a significant increase after 30 min. Amini et al. (2015) concluded that temperature was in fact more determinative in modifying starch solubility than ultrasonication time. These authors determined that S was almost independent of exposure time at temperatures below 45 °C, but at higher temperatures, a significant direct dependence was observed [13]. One possible explanation is that high temperatures cause more serious physical destruction within the granules and the unraveling of double helices of the crystalline region, which reduces the stability of starch [25,32]. Hou et al. (2023) also attributed the increase in SP and S to the cavitation-induced increase in temperature [77].

The susceptibility of SP and S to be modified by USC also depends on the type and composition of the starch, particularly the structural arrangement of amylose and amylopectin. Ultrasonication breaks the intermolecular bonds of starches, generating a less compact granular arrangement and resulting in an increase in apparent amylose content (i.e., a higher amount of short light chains), which allows a greater possibility to interact with water (increase in SP) and eases the release of amylose out of the granule to the aqueous medium (increase in S) [26,51]. Carmona-García et al. (2016) concluded that the granule size also positively influences the modification of SP and S since larger granules have a larger surface area prone to be affected by sonication [61].

### 5.2. Pasting Properties

Pasting properties quantify the viscosity development of starch in an aqueous solution as it is heated at a known rate, kept at a maximal temperature for a definite period and cooled down to allow amylose retrogradation, also at a known rate, all while intensely mixing. Under the mechanical shear applied by mixing, the polymers tend to align themselves, increasing the viscosity in a balanced way with granule swelling [9]. Pasting properties are influenced by the type of starch, structure of amylose and amylopectin and the presence of nonstarch components due to the interaction they may have with starch [10]. USC has different effects on the modification caused by pasting properties depending on the source of the starch and the treatment conditions applied. For instance, amylose content appears to be directly correlated with a higher susceptibility to modification, probably related to the easier mobility of amylose chains compared to amylopectin molecules [79].

The pasting temperature (PT) is the temperature at which viscosity begins to increase during heating, which indicates the structural resistance of starches to heat-induced swelling and rupture in water [9,30]. There is no consensus regarding the effect of USC on PT. Some authors have reported a PT increase after ultrasonication, which indicates better resistance to high temperatures and strong mechanical shearing force due to treatments [18,32,40]. An increase in PT supports the argument that ultrasonication tends to increase starch crystalline perfection, resulting from the reorientation of molecules or chains. The strengthening of intragranular bonded forces makes starch granules require more heat and a longer time before structural disintegration and paste formation occur [28,79]. When a significant lowering of PT has been reported, it has been indicated that ultrasonication caused granule disruption and partial degradation of amylopectin, which made them more permeable to water and less resistant to swelling [19,30].

Ultrasounds have generally been reported to decrease the viscosity achieved during pasting events in starches [26,32,72,79] and flours [53,65,73]. In flours, the modification caused to the viscometric profiles also depends on the effect of USC on other viscosity contributors such as proteins and fibers and the interactions that they may have with starch [9]. Lower viscosity profiles are attributed to both the physical damage caused to starch granules and to changes in the starch molecular structure [15,31]. Starch granule breakdown promotes the penetration of water while the degradation of macromolecular chains by sonication generates shorter chains, which would result in lower viscosity during gelatinization. The reduction in the molecular chain length and molecular weight by cavitation results in a weaker interaction force of starch granules and a partially degraded starch gel network that is less resistant to shear, resulting in lower viscosity profiles [40,42].

Because of the continuous swell of starch granules, peak viscosity (PV) can be acquired from a complete rupture of its inherent hierarchical structures [30]. The reduction in PV after sonication indicates that USC could weaken the integrity and rigidity of the granule structure due to glycosidic bond cleavage [19,48]. The lessened amounts of crystallites, amorphization of short-range ordered structure and disruption of helical structures caused by violent physical forces (e.g., micro jets, shear forces, shock waves) and -OH free radicals during ultrasonication might reduce PV as the disordered aggregation structures of starch often show weak resistance when subjected to shearing and heating [30,45]. The breakdown viscosity (BV) reflects the degree of granule disruption after reaching the maximum viscosity value. Lower BV values after ultrasonication suggest a stronger resistance of the starch granules to shear-thinning during cooking and strengthen stability in the hot paste [19,30,36]. The final viscosity (FV) indicates the ability of the sample to form a viscous paste or gel after heating and cooling [9], while the setback viscosity (SV) reflects the retrogradation capacity of starchy foods, positively correlated with amylose content [11,36]. Lower FV and SV values after USC are attributed to a decrease in the degree of polymerization of treated starches due to the degradation and depolymerization of the leached amylose and long-chain amylopectin [40,49]. A drop in SV values indicates that short-term retrogradation could be depressed under USC treatments [45].

It is worth mentioning that pasting viscosities have not always been reported to be reduced by ultrasonication since, as seen in other properties, the modification depends on the treatment condition array. Some authors have indicated no significant changes [9,39] or higher values after ultrasonication [33,47,67,75,76]. Increased pasting values have been attributed to (a) a possible loosening of the interaction between amylose and amylopectin chains after ultrasonication [47], (b) microcrystallization and reorientation of starch molecules or chains [33] and (c) a softer starch matrix induced by sonication resulting in an easier pasting and higher viscosity [76]. Herceg et al. (2010) said that increasing power (300 and 400 W) caused a greater disruption of starch granules and weakening of the crystalline region, entrapping more water within the starch molecule, which led to higher viscosity [1]. An increase in SV has been attributed to a higher amount of linear chains after degradation and depolymerization of long-amylose chains, facilitating the reassociation or rearrangement of starch molecular chains during cooling [18,30].

### 5.3. Paste Clarity

Starch paste clarity is an important quality parameter in food applications, as it influences the optical properties of final products [31]. The transmittance of starch pastes is associated with particle size distribution and the proportion of amylose and amylopectin. A larger expanded particle size and greater amylopectin content tend to increase starch paste transparency [8,74]. Ultrasounds can break amylopectin chains by disrupting covalent bonds, resulting in a decreased association between starch molecules and causing a paste clarity increase [8,74]. USC treatments led to increased paste clarity in potato [42], corn [7,74], sweet potato [8], maize [6], mung bean [80], millet [39] and rice [42] starches, which seems to be improved by longer sonication times [6]. The extent of the effect has been reported to depend on the sonication device used. At the same frequency, Jambrak et al. (2010) and Falsafi et al. (2019) reported a greater increase in transmittance when USC was applied using a probe than when a bath was used [7,31]. The solvent used for treatment also influences the effect of USC over paste clarity due to the medium’s surface tension and the dynamics of bubble formation and implosion. Sujka and Jamroz (2013) determined that potato starch’s paste clarity was increased after sonication in water but it was not modified when sonicated in ethanol [42]. Increased paste clarity probably results from damage caused by USC to the starch surface and the crystalline regions, leading to an increased association between starch and water (thereby resulting in a decreased association between starch molecules) making starch particles expand easily and increasing the paste transparency [74]. However, some authors have indicated no significant change [18] and even a paste clarity reduction [47] after ultrasonication at strong USC conditions. A clarity decrease has been attributed to starch granule disintegration, allowing them to swell more, making the starch paste more viscous and decreasing the transmittance [18]. It could also be attributed to the degradation of amylose and amylopectin and the fissures formed on starch granules, favoring the essential linear amylose molecule to leach out of the granule, hence reducing paste clarity [47].

## 6. Effect of Ultrasonication on the Thermal Properties

The onset (*T_O_*) and conclusion (*T_C_*) temperatures reflect the melting temperatures of the weakest crystallites and high-perfection crystallites in the starch granules, respectively [36], while the gelatinization temperature range (Δ*T* = *T_C_* − *T_O_*) represents the extent of homogeneity of crystallites within the granules [31]. It has been said that power, time and temperature highly influence the modification caused by ultrasonication to gelatinization temperatures [13,23,39,41,45]. Some authors have indicated that USC does not lead to significant changes in gelatinization temperatures [7,44,52], while most of the literature has reported significant modifications. A significant shift toward higher temperatures has been reported in a wide variety of starches such as maize [26], corn [13,37], rice [76], yam [47], oat [31], waxy corn [45], potato [33,71] and cowpea [72] and potato flour [77]. The weakest crystallites are more prone to be disrupted during sonication, resulting in a delayed *T_O_* after treatments, indicative that the crystalline structure in the USC-treated sample requires higher temperatures to be dissociated [26,45,71,76]. On the contrary, a significant reduction in gelatinization temperatures has been reported after ultrasonication of rice [23], maize [81], corn [19], millet and potato [32], chestnut [36], pea [63] and sweet potato [30] starches as well as in quinoa [65] and brown rice [55] flours. It was found by Yu et al. (2013) that *T_O_* and *T_P_* values showed an inverse correlation with the applied USC power, which could explain the different results reported in the literature [23]. Lower gelatinization temperatures could be caused by a change in the starch matrix due to a greater mobility of starch polymers after ultrasonication, promoting water entering the granules and accelerating the hydration process [32]. Despite the different results reported for *T_O_*, *T_P_* (peak temperature) and *T_C_* after USC treatments, there seems to be a general agreement that Δ*T* is significantly reduced after ultrasonication, particularly when applying high USC intensity [31,47] or high temperature [13,25]. The narrowing of Δ*T* may happen because USC disrupts the ordered double-helical structures containing flaws and leads to the breakage of crystallites of different stabilities, decreasing the degree of diversity in the crystals and lowering the dissociation temperature range [26,30,43]. Said narrowing has also been explained as the distortion of the amorphous and nonorganized parts of the starch granule by ultrasonication, which might enhance the homogeneity of starch granular structure toward a well-ordered crystalline remnant with narrower Δ*T* [13].

The gelatinization enthalpy (Δ*H*) values reflect the loss of double-helical order in the crystalline and noncrystalline regions of the granules that unravel and melt during starch gelatinization [39,49]. The effect of USC on Δ*H* depends greatly on the treatment conditions and the sample’s botanical origin (amylose/amylopectin composition). Yang, Kong et al. (2019) and Yu et al. (2013) determined that the change is influenced by the applied power, where low power USC (≤300 W) leads to reduced Δ*H* values, while an increase in Δ*H* was reported when applying higher powers (≥450 W) [23,40]. Higher Δ*H* denotes a greater number of double helices with more compact packing (indicating that the amorphous regions were degraded by USC prior to the crystalline regions) [38,39], a rearrangement of the molecular packing within the granule microstructure [37,61] and that ultrasonicated starches presented a higher amylopectin content resulting from leaching of amylose in the liquid medium where treatment was performed [55]. The amylose/amylopectin composition of the treated matter and the degree of damage that its amorphous and crystalline regions suffer due to USC also determines the final effect over Δ*H*. With this complex array of variables, it is easy to understand why there is not a consensus in the literature regarding the effect of USC treatments on Δ*H*. It is worth mentioning, however, that in general, more intense USC conditions modify the crystallinity of the starch and reduce Δ*H* [79].

A decrease in Δ*H* after ultrasonication has been attributed to (i) surface damage caused to starch granules, increasing the access of water molecules to the crystalline regions [40]; (ii) disintegration of the double helices present in the crystalline and noncrystalline regions of the granule by cavitation [15,26,38]; and (iii) distortion of the crystalline regions by H^+^ ions released during the sonochemical ionization of water molecules [31]. Δ*H* has been positively correlated to the branch-chain length of amylopectin. A decrease in Δ*H* indicates that some of the external chains of amylopectin are destroyed after ultrasonication [40,45] so the USC-treated starch would require less energy for gelatinization [10], in agreement with effects reported over long-range and short-range ordered structures [30].

Some authors have evaluated starch retrogradation properties with a second scan of the gelatinized starch after storage of the samples, which usually refers to amylopectin retrogradation Ultrasounds have been reported to have little effect on retrogradation temperatures [23] and enthalpy [10]. It was indicated by Yu et al. (2013) that the effect of retrogradation enthalpy depended on the applied USC power. These authors observed that retrogradation enthalpy decreased slowly in samples treated at 100 and 500 W but a sharp decrease was determined when the power was 1000 W. High USC power can destroy the highly branched structure of amylopectin and degrade its molar mass, so much less amylopectin would be recrystallized, leading to lower retrogradation enthalpy values [23].

## 7. Effect of Ultrasonication on Gel Properties

Starch finds many industrial applications mainly due to its gelling capacity when heated in presence of water [1]. In the gelatinization process, linear and short-chain amylose and amylopectin chains are leached out from the granules, forming a highly viscous continuous matrix, while amylopectin-rich insoluble remnants are dispersed in the continuous matrix, forming a microstructure with complex rheological response [37]. The rheological behavior and textural properties of the gels depend strongly on the starch molecular structure [37], so the fragmentation of amylose chains and debranching of amylopectin molecules by ultrasounds also modify the properties of the gels formed with the USC-treated starches and flours.

### 7.1. Rheological Properties

Rheology reflects the flow and deformation behaviors of fluid foods, which are typically determined by processing conditions such as high shearing, stirring, mixing and pumping. The rheological behavior of starch gels is considered one of the most important physical properties for determining the eating quality of starch or starch-based foods and their acceptance by consumers [30]. The rheological properties are strongly dependent on the accessibility and entanglement of amylose and amylopectin, chain length distributions and ramification degree of leached starch chains, the interactions and tightness between polymer chains and the dissolution ability of amylose, which determine the stability of the formed three-dimensional network structure [30,37].

#### 7.1.1. Steady Shear Flow Behavior

The flow behavior of starch gels is analyzed by measuring their apparent viscosity over a defined shear rate range (at constant temperature), usually upward and downward, to later apply least-squares fitting to a power-law model that describes the flow behavior of pseudoplastic fluids, the most common among starch gels in a relatively dilute medium. The flow behavior index (*n*), the consistency coefficient (*k*) and the time-dependence behavior are determined with these models. Rotational tests allow us to analyze the rheological behavior of the gels under large shearing deformations [52].

**Figure 9:**
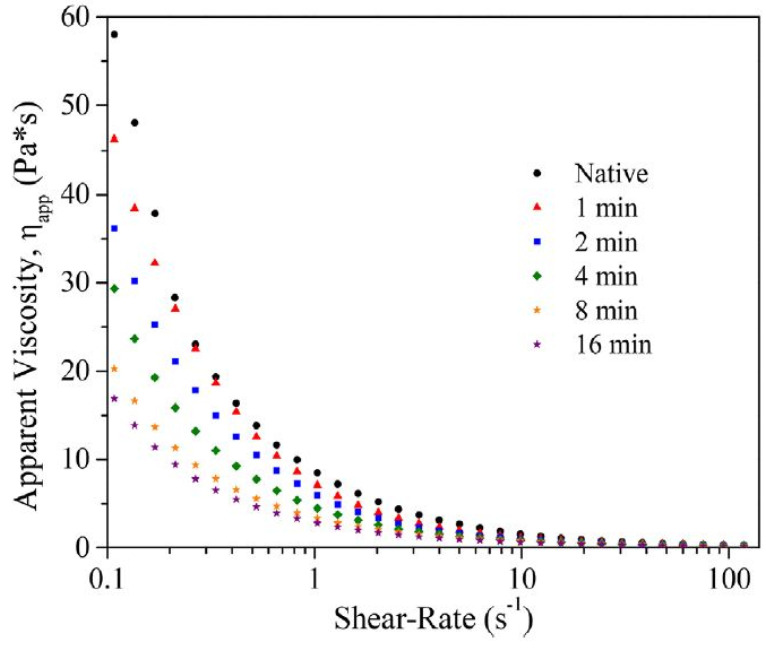
Behavior of the apparent viscosity of native corn starch and corn starch sonicated for different times [37].

It has been indicated that the apparent viscosity of starch gels is reduced by ultrasonication due to the granular structure destruction and depolymerization of the main components (fragmentation of amylose chains and debranching of amylopectin molecules) caused by cavitation in C-O-C linkages [5,13,80]. Flores-Silva et al. (2017) determined decreasing apparent viscosity values with longer sonication times (see Figure 9), indicative of weaker gels that reflect the fragmentation of long-chain starch molecules [37]. Kang et al. (2016) also reported a sharp apparent viscosity decrease at the early stage of sonication and then a slower reduction reaching a limiting value [5]. This slowdown in the rate of apparent viscosity reduction reflects how starch chains become shorter with increasing ultrasound exposure and progressively approach the minimum chain length that limits the USC degradation process [7], first mentioned by Czechowska-Biskup et al. (2005) [16].

Both *n* and *k* have been reported to be modified by ultrasonication at different extents. The starch concentration and sonication amplitude do not appear to have a significant influence on the value of *n* [13] while the time and temperature highly influence the effect of USC on *n*. Starches usually present values of *n* < 1, displaying a pseudo-plastic behavior [71]. Kang et al. (2016) obtained an increase of *n* with sonication time, indicating the formation of weaker gels as the starch gels were subjected to longer USC exposure, diminishing pseudoplasticity to the point where gels showed a Newtonian behavior (*n* = 1) [5]. These results could be due to solubilization (disaggregation) of the aggregated macromolecules in the starch pastes by ultrasonication, so the shear applied during viscosity measurements will no longer contribute to the break-down of the starch aggregates, resulting in a reduction in the thixotropic behavior [5]. Starch molecular size reduction will also result in the transition from a pseudoplastic to a Newtonian behavior, characteristic of a dilute macromolecular suspension [5]. Amini et al. (2015) determined that the effect of ultrasonication on *n* depends highly on the interaction between the treatment temperature and time. At temperatures close to 45 °C, the sonication time did not affect *n* but at lower temperatures, increasing the sonication time decreased the value of *n*. In contrast, increasing the exposure time at higher temperatures (>45 °C) caused a profound *n* increase [13]. Zhang et al. (2021) agreed on this in the study where the ultrasonication of corn, potato and pea starches was performed at different times and a constant temperature (25 °C), where *n* values were reduced [71].

Regarding *k*, the available literature suggests that temperature is the main variable determining the modification caused by ultrasonication. Amini et al. (2015) showed that *k* in corn starch increased with increasing temperature up to 45 °C while increasing the temperature to 65 °C significantly decreased *k* [13]. About the evolution of *k* in ultrasonicated corn, Zhang et al. (2021) concluded that USC treatments at different powers (100–600 W) and times (5–30 min) caused an increase in *k* values, while Jambrak et al. (2010) found that *k* values were reduced after ultrasonication in similar power (100–400 W) and time (15 and 30 min) ranges [7,71]. The different results are believed to derive from the treatment temperature. In the treatments performed by Zhang et al. (2021), it is indicated that the temperature was 25 °C, which lies in the temperature range where Amini et al. (2015) said that ultrasonication causes an increase of *k* (<45 °C). In the study carried out by Jambrak et al. (2010), however, the treatment temperature was not indicated, which usually means that it was not controlled, so the treatment temperature would have presumably risen beyond 45 °C, explaining the *k* reduction following the explanation made by Amini et al. (2015). In the cases where Jambrak et al. (2010) reported an increase of *k* (treatments applied using USC bath, and one treatment using a probe at 100 W for 15 min), it is believed that ultrasonication conditions were rather soft (low power and/or short time), so the treatment temperature may not have been significantly increased, possibly lying in the <45 °C range. Higher *k* values suggest gels with high structural strength and resistance to flow, which might result from the loose internal crystalline structure of sonicated corn starch [71]. The alteration of these rheological parameters may be attributed to a combined effect of starch granule disruption and breakdown of the linear amylose molecules [13].

#### 7.1.2. Dynamic Oscillatory Assays

##### Strain/Stress Sweeps

Strain or stress sweeps at a constant angular frequency are performed to determine the limit of the linear viscoelasticity of the gels [30]. These assays indicate two different regions in gel behavior, the linear viscoelastic region (LVR), where the elastic (*G*′) and viscous (*G*″) moduli as well as the loss tangent [*tan(δ)*] are constant, and the nonlinear region, where the gels quickly lose their structure’s integrity. The maximum deformation that gels resist before the disruption of their structure is denoted by τ_max_, which marks the end of the LVR [25]. These tests have been used by some authors [13,51,52,61] when studying USC-treated starches to determine the LVR in order to carry out frequency sweeps. However, there have not been reported results regarding the effect of USC treatments on the maximum stress the gels can stand before breaking their structure (τ_max_) or the cross-over point (*G*′ = *G*″). On USC-treated flours, it has been indicated that ultrasonic treatments ≥ 10 min led to an increase in τ_max_ in rice [25,53] and tef [24] flours. These results suggest that USC treatments can lead to stronger gels that resist higher stress before the rupture of their structure [53]. Ultrasonication of rice flour at higher temperatures (50 and 60 °C) led to a significant reduction in τ_max_, as well as a progressive cross-over point decrease with increasing temperature, indicative of weaker gel structures due to the damaging effect of high temperatures [25]. Regarding the composition of the flour, it has been indicated by Náthia-Neves et al. (2024) that the lipid content would also influence whether USC leads to a reduction or increase in τ_max_ after finding a 62% reduction in whole canary seed flour and a 32% increase in the deffated counterpart sonicated under the same conditions [73]. There is still more research needed to reach more solid conclusions on the effect of USC treatment on strain/stress sweeps parameters.

##### Frequency Sweep

Frequency sweeps are performed on gels to determine their frequency dependence at a specific temperature, applying a constant strain or stress (within the LVR). The viscoelastic properties of gels as a function of frequency are characterized by storage (*G*′) and loss (*G*″) moduli and loss tangent [*tan(δ)* = *G*″/*G*′], which are used to quantify the strength of gels [13]. Higher values of *G*′ than *G*″ show a predominance of the solid/elastic behavior [51]. The modification that USC treatments cause to the frequency sweep parameters is influenced by starch granule disruption and the breakdown of amylose chains [13]. There seems to be a general agreement that the macromolecular fragmentation induced by ultrasonication leads to short polymeric chains in USC-treated starches. However, there is no consensus regarding the association that the shorter chains may have during gelatinization, and while some sources report increased moduli after treatments, others indicate the contrary. The treatment time seems to be the main factor influencing the extent of modification achieved in frequency sweep parameters. Short treatment times have been reported to increase moduli, presumably due to the weakening of the crystalline regions, causing the molecules to entrap more water, resulting in higher viscosity [51]. Within the short time range (≤20 min), Monroy et al. (2018) concluded that increasing the time leads to stronger gels (higher *G*′ values) as fragmentation of starch polymer chains facilitates the gelation process and subsequent retrogradation by reassociating short polymer chains through hydrogen bonding to form a more elastic three-dimensional network [52]. Zhang et al. (2021) reached a similar conclusion after ultrasonicating corn, potato and pea starches at different power levels and times. These authors attributed the increases in *G*′ and *G*″ to the degradation of starch granules, making them more permeable to water, and the tendency of starch molecules (especially amylose) to reform double helices resulting in harder gels [71]. However, at longer times (30 min according to Kaur and Gill (2019)), it has been indicated that both moduli are reduced, attributed to the severe damage caused to the starch granules by the shear forces of cavitation, leading to the straightening out of amylose molecules that reduces the shear action within the fluid layers and contributes to a viscosity decrease. A sharp decrease in *G*′ was determined by Carmona-García et al. (2016) after 50 min ultrasonication of plantain and taro starches, suggesting that amylopectin was largely affected by treatment, which generated linear chains that were not able to form a consolidated compact network during gelatinization [61]. A significant decrease in *G*′ and *G*″ has also been reported in rice flour ultrasonicated for 60 min at different temperatures [25], while only *G*″ was reduced when applying USC at shorter times [53].

Loss tangent (*tan(δ)*) gives information about the relative importance of the viscous versus elastic components of the viscoelastic behavior of the gel, which indicates the ratio of energy lost to the energy stored (*G*″/*G*′). Despite the different results reported for *G*′ and *G*″ depending on the applied time, the literature seems to agree about the lowering of *tan(δ)* after ultrasonication, suggesting that USC could change the state of starch pastes to more solid-like behavior [71]. Lower *tan(δ)* values have been indicated for longer treatment times, while at shorter times, only slight variations (or even no changes at all) have been reported [13,51,61]. Amini et al. (2015) obtained a *tan(δ)* increase in corn starch ultrasonicated at 65 °C (beyond the onset temperature of gelatinization, *T_O_*, of the native starch), which must be caused by the high temperature and not by ultrasonication, given that treatment at lower temperatures did not show the same effect [13]. In the ultrasonication of rice flour at 60 °C (below *T_O_* of the native flour) Vela, Villanueva and Ronda (2021) still determined a reduction in *tan(δ)*, indicative that *T_O_* could mark the limit of sonication temperature that would lead to lower values of *tan(δ)* [25]. When there is a rise in temperature, the molecules absorb translational energy and gradually cease to retain their hydration, which causes the lowering of viscosity [51]. The effect of ultrasonication in reducing *tan(δ)* could be attributed to structural rearrangement, straightening out of amylose and disruption of starch granules in sonicated samples [71]. It has been said that ultrasonicated starches could be used as strong gelling agents in the food industry given the higher elastic behavior of gel showed by *tan(δ)* [30].

### 7.2. Textural Properties

Gel’s texture profile is usually characterized by hardness, cohesiveness, adhesiveness, springiness, gumminess and chewiness, usually established by instrumental assays [28]. Gel hardness is mainly caused by retrograding starch [1]. The available literature has demonstrated that ultrasonication led to lower hardness in gels prepared from corn, [74], oat [31], potato [82], foxtail millet starches [28] and quinoa [65], wheat, purple dawn sweet potato and red sweet potato flours [10]. Adhesiveness [10], cohesiveness, springiness [28,65], elasticity, conglutination degree, chewiness and recoverability [74] have also been reported to be reduced by ultrasonication. The intramolecular hydrogen bonding of starches can be broken due to mechanical vibration, thermal and ultrasonic effects, thus the molecular structures become loose and molecular winding nodes are reduced [74]. Larger hardness reductions have been reported for higher frequencies and longer sonication times since starch granules become further disrupted and depolymerized by greater sonication exposure [10,28]. The liberation of low molecular weight short glucan fractions from amylose and amylopectin following cavitation reduces the ability of starch to form homogenous gel structures. Chains with a polymerization degree below 12 negatively affect the gel formation ability of starches [31]. In flours, the weaker gelation could also be a consequence of the degradation of pectins and sugars by ultrasonication, affecting their cross-linking with starch [10].

However, some authors have reported that the disruption and depolymerization of starch granules lead to higher values of hardness, adhesiveness, cohesiveness, springiness and gumminess on gels made with USC-treated corn [1], kiwi [58], maize and quinoa [70] starches. The common elements in these papers are the high-power levels and long times used for ultrasonication (see details in Table S1). The combination of long exposure to high power and low starch concentration in the aqueous dispersion may lead to an annealing effect, even when temperature is controlled. This combined annealing-ultrasonic treatment would lead to significantly different results than what is obtained only by USC [13,25,32]. In the studies presented by Babu et al. (2019) and Amarnath et al. (2023) comparing the texture properties obtained in gels made from millet starches modified by USC and annealing treatments separately and by a combination of both (annealing + ultrasound and ultrasound + annealing), the authors determined that the application of ultrasound alone decreased the hardness of the gel but the application of annealing increased the hardness [27,28]. Sonication damaged starch granules, causing amylose leaching and cutting long internal chains into appropriate length ones, which allow them to participate in network formation during the annealing and retrogradation process, forming harder gels [28,70]. It has been said by Su et al. (2024) that the high USC power has to be within a defined power range to enhance the hardness, consistency and cohesiveness of the starch gel (up to 600 W in their study), since exceeding this range would cause excessive disruption and damage to the granules (found at 900 W for purple rice starch), leading to decrease in texture parameters [34].

## 8. Effect of Ultrasonication on Starch Digestibility

Starch in vitro digestion properties are commonly evaluated following the procedure of Englyst et al. (1999), which simulates human gastrointestinal tract digestion, where starch samples are incubated with pancreatin from porcine pancreas and amyloglucosidase enzymes at 37 °C for 120 min [83]. For nutritional applications, starch comprises three portions: rapidly digestible starch (RDS) (the fraction digested within 20 min), slowly digestible starch (SDS) (the fraction digested between 20 and 120 min) and resistant starch (RS) (the remaining fraction after 120 min) [10,71]. RDS is digested in the mouth and small intestine, resulting in a rapid increase in postprandial blood sugar level, whereas SDS is slowly digested in the small intestine, leading to stable postprandial blood sugar levels [71]. RS is not digested in the small intestine and does not contribute to blood glucose levels, becoming a substrate for the intestinal microbiota, thus providing health benefits and reducing risk factors for diet-related diseases [43,71]. Starch digestion properties can be evaluated in raw and in gelatinized starches; the former gives insight about the starch structure and its accessibility to digestive enzymes and the latter reflects the impact that the sample would have in the final product, once cooked, where gelatinized samples present lower values than their raw counterparts due to cooking [10,51].

Some authors have not reported differences in RDS after ultrasonication [29,37,72], while others reported an RDS increase [10,33,51,63,67] or reduction [58,77]. The RDS content of different USC-treated starches (wheat, barley, rice and maize) increased with increasing sonication time, both in raw and gelatinized samples [51]. The same trend was reported in sonicated flours (purple dawn sweet potato, red sweet potato, wheat and quinoa) [10,65]. This direct correlation of increasing RDS values with sonication time could be explained by the holes and cracks formed on granules due to cavitation. Crystalline regions are more compactly arranged than amorphous regions and thus less susceptible to attack by digestive enzymes but after sonication, the double-helix structure of starches might be disrupted, allowing easier access to enzymatic hydrolysis [10,37,51]. The RDS increase in gelatinized starch might also be explained by the increased availability of short-length chains obtained from the fragmentation of amylose chains and debranching of amylopectin molecules, which are more amenable for enzymatic degradation [37]. In the case of SDS, there is not a clear effect of USC treatments. While some studies have reported a significant decrease [29,33,51], others indicated an increase after ultrasonication [10,72,77]. RS content has been said to increase after USC treatments [28,29,33,51,58,77], possibly due to interactions between different chains of starch or microconstituents (such as amylose and amylopectin, amylose and protein, amylopectin and lipid, etc.) and the rearrangement and formation of hydrogen bonds within the starch inner structure [33]. Said increase, however, would only happen at low-power ultrasonication. Zhang et al. (2021) and Hou et al. (2023) reported that RS content increased significantly at USC treatments up to 300 W, while higher USC powers (400–600 W) led to an RS content reduction in corn starch and potato flour, respectively [71,77]. It is believed that sonicating starch at low power led to a rearrangement of starch molecules that reduced its susceptibility to enzyme hydrolysis, while higher power caused more serious physical destruction within the starch granules and broke the crystalline molecular structure to a state that was easily accessible to enzymes, resulting in a decrease in RS content. These results suggest that the rearrangement and destruction of starch structures occur simultaneously during USC treatment and that the destruction plays the dominant role at high-power USC [71]. The treatment temperature has also been indicated to influence the modification caused to RS. In ultrasonication of corn starch, Ouyang et al. (2021) found that treatments performed at a low temperature (≤25 °C) led to higher RS content, while increasing the temperature to 35 and 50 °C decreased the RS content. It was indicated by the authors that the gelatinization process that happens at higher-temperature USC (35, 50 °C) disrupted the original helical and crystalline structures in corn starch while the retrogradation process observed at lower-temperature treatments (≤25 °C) promoted the reassociation of ordered structures that eventually transformed part of SDS and/or RDS into RS [41]. The evolution of RS after USC treatments could also depend on the nature and state of the sample during in vitro assays. While Kaur and Gill (2019) reported that RS significantly increased with longer ultrasonication in both raw and gelatinized starches, Cui and Zhu (2020) found that RS in raw flours was reduced by ultrasonication but when analyzing gelatinized flours, RS showed increased values with increasing times [10,51]. The different results between both studies could derive from the fact that Kaur and Gill (2019) worked with starches while Cui and Zhu (2020) worked with flours. Ultrasound could have also weakened starch–protein integrations, increasing starch hydrolysis in flour samples [10].

**Figure 10:**
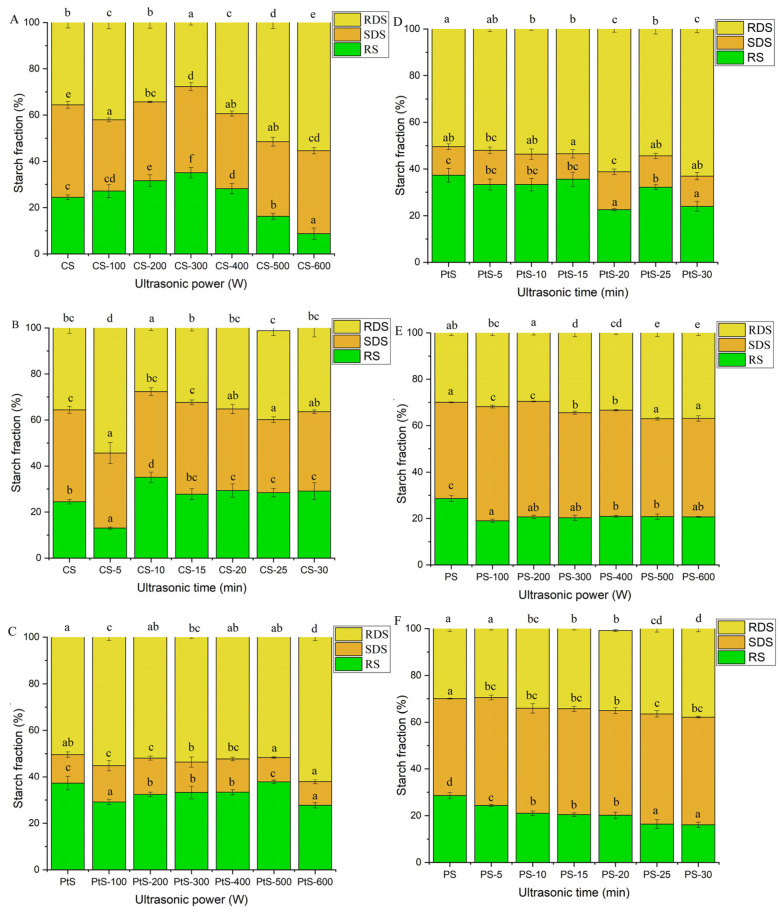
Digestion profile of native and sonicated corn, potato and pea starches under different ultrasonic powers and times; (**A**,**B**) native and sonicated corn starch (CS); (**C**,**D**) native and sonicated potato starch (PtS); (**E**,**F**) native and sonicated pea starch (PS). Different letters upon the pillars of the same color indicate statistical differences (*p* < 0.05) [71].

The effect of USC on starch digestion properties is complex and depends on the applied treatment parameters, such as time [37], power [71], temperature [41], starch botanical origin [71] and even granule size [51] (see Figure 10). The granule size may also affect starch digestibility since smaller-sized starches exhibit a higher digestibility rate due to their increased surface area for specific volume, which increases the chance of enzymatic attack on substrate [51]. Further research is needed to reach solid conclusions regarding the effect of USC on starch digestion properties.

**Table 1:** Summary of the available literature about modifications of starches, flours and grains using ultrasonic treatments.

Botanical Origin	Parameters Studied	Reference
**STARCHES**		
Potato	Frequency, time, atmosphere, concentration, volume	(Gallant et al., 1972) [21]
	Frequency, time, atmosphere, concentration, volume	(Degrois et al., 1974) [62]
	Time, temperature, concentration	(Azhar and Hamdy, 1979) [84]
	Time, source of starch	(Chung et al., 2002) [80]
	Source of starch, USC application device, concentration	(Iida et al., 2008) [4]
	Source of starch, solvent	(Chan et al., 2010) [48]
	Power	(Zhu et al., 2012) [46]
	Power	(Zuo et al., 2012) [20]
	Solvent, source of starch	(Sujka and Jamroz, 2013) [42]
	Frequency, power, time	(Bai et al., 2017) [2]
	Source of starch, solvent	(Sujka, 2017) [3]
	Frequency, temperature	(Hu et al., 2019) [32]
	Concentration	(Nie et al., 2019) [82]
	Single and dual treatments	(Cao and Gao, 2020) [33]
	Temperature	(Ouyang et al., 2021) [41]
	Power, time	(Zhang et al., 2021) [71]
	Single and dual treatments	(Wang, Wang et al., 2022) [35]
	Single and dual treatments	(Zhou et al., 2023) [85]
Waxy potato	Frequency, power, time	(Bai et al., 2017) [2]
Sweet potato	Time, temperature, concentration	(Azhar and Hamdy, 1979) [84]
	Source of starch, USC application device, concentration	(Iida et al., 2008) [4]
	Frequency, time	(Zheng et al., 2013) [8]
	Intensity, time, temperature, concentration	(Jin et al., 2020) [49]
	Time	(Wang, Xu et al., 2020) [30]
	Time, temperature	(Ulfa et al., 2023) [86]
Rice	Time, source of starch	(Chung et al., 2002) [80]
	Solvent, source of starch	(Sujka and Jamroz, 2013) [42]
	Source of starch, solvent	(Sujka, 2017) [3]
	Source of starch, time	(Kaur and Gill, 2019) [51]
	Power	(Yang, Kong et al., 2019) [40]
	Dual treatments	(Li et al., 2022) [87]
	Probe tip, power, time	(Yu et al., 2013) [23]
Purple rice	Single and dual treatments, poser	(Su et al., 2024) [34]
Waxy rice	Time, temperature	(Isono et al., 1994) [69]
	Source of starch	(Luo et al., 2008) [26]
	Power, intensity, temperature	(Zuo et al., 2009) [15]
Corn/ Maize	Atmosphere	(Czechowska-Biskup et al., 2005) [16]
	Time	(Huang et al., 2007) [38]
	Source of starch, USC application device, concentration	(Iida et al., 2008) [4]
	Source of starch, solvent	(Chan et al., 2010) [48]
	USC application device, power, time	(Herceg et al., 2010) [1]
	USC application device, power, time	(Jambrak et al., 2010) [7]
	Solvent, source of starch	(Sujka and Jamroz, 2013) [42]
	Frequency, time	(Hu et al., 2014) [74]
	Amplitude, time, temperature, concentration	(Amini et al., 2015) [13]
	Frequency	(Hu et al., 2015) [56]
	Power, time, concentration	(Kang et al., 2016) [5]
	Time	(Flores-Silva et al., 2017) [37]
	Source of starch, solvent	(Sujka, 2017) [3]
	Power, time, temperature	(Li et al., 2018) [19]
	Source of starch	(Minakawa et al., 2019) [64]
	Temperature	(Ouyang et al., 2021) [41]
	Time	(Rahaman et al., 2021) [44]
	Power, time	(Zhang et al., 2021) [71]
	Dual treatments, time	(Yilmaz and Tugrul, 2023) [88]
	Source of starch	(Luo et al., 2008) [26]
	Time	(Bel Haaj et al., 2013) [6]
	Dual treatments, time	(Flores-Silva et al., 2018) [81]
	Source of starch, time	(Kaur and Gill, 2019) [51]
	Single and dual treatments	(Wang, Wang et al., 2022) [35]
	Source of starch, time	(Wei et al., 2023) [70]
	Single and dual treatments	(Zhou et al., 2023) [85]
Waxy corn/maize	Source of starch, USC application device, concentration	(Iida et al., 2008) [4]
	Time	(Bel Haaj et al., 2013) [6]
	Time	(Wei et al., 2021) [78]
	Power	(Yang, Lu et al., 2019) [45]
High-amylose maize	Glycerol content	(Lima and Andrade, 2010) [89]
Amylomaize V	Source of starch	(Luo et al., 2008) [26]
Tapioca/cassava	Source of starch, USC application device, concentration	(Iida et al., 2008) [4]
	Amplitude, time	(Manchun et al., 2012) [59]
	Amplitude, time, temperature	(Monroy et al., 2018) [52]
	Source of starch	(Minakawa et al., 2019) [64]
	Time	(Rahaman et al., 2021) [44]
Wheat	Time	(Seguchi et al., 1994) [68]
	Solvent, source of starch	(Sujka and Jamroz, 2013) [42]
	Source of starch, solvent	(Sujka, 2017) [3]
	Source of starch, time	(Kaur and Gill, 2019) [51]
	Source of starch, time	(Karwasra et al., 2020) [50]
Barley	Source of starch, time	(Kaur an Gill, 2019) [51]
Oat	USC application device, time	(Falsafi et al., 2019) [31]
Millet	Frequency, temperature	(Hu et al., 2019) [32]
	Frequency, time, concentration	(Li et al., 2019) [39]
White finger millet	Single and dual treatments	(Amarnath et al., 2023) [27]
Foxtail millet	Single and dual treatments	(Babu et al., 2019) [28]
Quinoa	Source of starch, time	(Wei et al., 2023) [70]
Taro	Amplitude, time, cycle	(Sit et al., 2014) [18]
	Time, source of starch	(Carmona-García et al., 2016) [61]
	Dual treatments	(Thomaz et al., 2020) [79]
Purple taro	Amplitude	(Martins et al., 2020) [90]
Yam	Amplitude, time	(Bernardo et al., 2018) [47]
	Source of starch	(Minakawa et al., 2019) [64]
Arrowhead	Tri-frequency treatments, power, time	(Raza et al., 2021) [57]
	Dual frequency, complex-formation	(Raza et al., 2023) [91]
Plantain	Time, source of starch	(Carmona-García et al., 2016) [61]
Sago	Source of starch, solvent	(Chan et al., 2010) [48]
Pinhão	Modification method	(Gonçalves et al., 2014) [92]
	Single and dual treatments	(Pinto et al., 2015) [75]
Chestnut	Single and dual treatments	(Wang, Wu et al., 2020) [36]
Mung bean	Time, source of starch	(Chung et al., 2002) [80]
	Source of starch, solvent	(Chan et al., 2010) [48]
Pea	Single and dual treatments	(Han et al., 2021) [67]
	Temperature	(Ouyang et al., 2021) [41]
	Power, time	(Zhang et al., 2021) [71]
	Temperature	(Han et al., 2023) [60]
	Single and dual treatments	(Hu et al., 2023) [63]
Cowpea	Dual treatments	(Acevedo et al., 2022) [72]
Banana	Amplitude, time	(Orsuwan and Sothornvit, 2015) [93]
	Amplitude	(Sun et al., 2022) [29]
Kiwi	Power, time	(Wang, Lv et al., 2022) [58]
Retrograded starch (RS3)	Power	(Ding et al., 2019) [43]
**FLOURS**		
Rice	Time, concentration	(Vela, Villanueva, Solaesa, et al., 2021) [53]
	Temperature	(Vela, Villanueva and Ronda, 2021) [25]
	Water removal method	(Vela, Villanueva, Náthia-Neves, et al., 2023) [66]
Corn	Water removal method	(Vela, Villanueva, Náthia-Neves, et al., 2023) [66]
Potato	Power, time	(Hou et al., 2023) [77]
Purple dawn sweet potato	Source of flour, time	(Cui and Zhu, 2020) [10]
Red sweet potato	Source of flour, time	(Cui and Zhu, 2020) [10]
Wheat	Source of flour, time	(Cui and Zhu, 2020) [10]
Tef	Temperature	(Vela, Villanueva, Li, et al., 2023) [54]
	Temperature	(Vela, Villanueva, Ozturk, et al., 2023) [24]
	Water removal method	(Vela, Villanueva, Náthia-Neves, et al., 2023) [66]
Canary seed	Temperature	(Náthia-Neves, et al., 2024) [73]
Quinoa	Time	(Zhu and Li, 2019) [65]
	Water removal method	(Vela, Villanueva, Náthia-Neves, et al., 2023) [66]
**GRAINS**		
Rice	Temperature	(Cui et al., 2010) [55]
	Time, temperature	(Park and Han, 2016) [76]
	Source of rice, amplitude, time	(Shah et al., 2023) [94]
Buckwheat	Solvent, sonicated amount	(Harasym et al., 2020) [9]

## 9. Conclusions

Ultrasound has proven to be a novel, fast and clean technology for modifying the structure and properties of starches and flours in order to expand their industrial applications. A complex set of variables, such as the ultrasonic equipment used, the treatment conditions, the solvent used, the suspension concentration, the drying method and the natural characteristics of the source under study, determine the modification that USC can produce on the treated material.

Cavitation causes granule damage in the form of holes, cracks and roughness and, in some cases, particle fragmentation, which increases the granules’ surface area, increasing their interaction with water and facilitating enzymatic reactions. At the molecular level, ultrasonication can cause partial depolymerization of starch components to varying degrees depending on the USC exposure. It is believed that USC preferentially degrades the linear amylose chains of the amorphous regions, probably because of their easier mobility while the highly branched amylopectin molecules require more energy during treatment to be disrupted (higher power and/or longer time). The molecular scission of the amylose chains and amylopectin branches increases the amount of linear fragments, resulting in a decrease in the starch molecular weight and an increase in apparent amylose content after treatments. The USC-modified starch structures show changes in short-range and long-range molecular order as a consequence of the structural rearrangement of starch molecules, which have been commonly reported to have greater homogeneity of crystallites.

The increased interaction with water after USC treatments results in the modification of the water-dependent properties, including pasting, rheological and gel textural properties of starches and flours. Lower *tan(δ)* values after ultrasonication have been attributed to enhanced interactions of amylose and amylopectin, leading to improved gel network structures. Resistant starch has been reported to be increased at low-power and low-temperature ultrasonication (<300 W).

The available literature indicates that the most important variables leading to more pronounced modifications in USC treatments are the botanical origin of the sample, USC power, time and temperature and the interaction between them due to increased damage caused by greater sonication exposure. When ultrasonication is applied at higher temperatures, the modification results in a combined ultrasonic-annealing treatment, which leads to significantly different results than those obtained by USC alone. Molecular rearrangement and destruction of starch structures occur simultaneously during USC treatments and the degree of modification achieved is determined by the combination of treatment factors. Further research is needed regarding the effect of ultrasonic treatments on the other components of flours (i.e., proteins, fibers, lipids), as the available literature focuses almost exclusively on the effects on starch.

The current trend in the modification of starches by ultrasonication is to combine them in dual treatments with other physical modification technologies (dual USC frequency, heat-moisture treatments, annealing, electric field, microwave) with the objective of enlarging the effect that USC can generate. A future interest is the scalability of this technology to an industrial level. The main challenge to do so is the need to eliminate a high amount of water after treatment, which most likely would be carried out by centrifugation of the dispersion and further removal of the supernatant (water containing the soluble compounds of the treated matter). The particle size reduction, starch fragmentation and modification of the rheological properties toward a more resistant structure present a promising improvement in the starches and flours, given that their main use is as a food ingredient (thickening, gelling agent, bulking agent, water retention agent and adhesive) and in baking, where the interaction that particles have with water and yeast can determine their applicability and suitability for many processes.

## Data Availability

No new data were created or analyzed in this study. Data sharing is not applicable to this article.

## Supplementary Materials

The following supporting information can be downloaded at: https://www.mdpi.com/article/10.3390/foods13152325/s1, Table S1: Detailed summary of the available literature regarding the modification of starches, flours, and grains by ultrasonic treatments.
