# Supplementary material for: Ultrasonication: An Efficient Alternative for the Physical Modification of Starches, Flours and Grains
Source: Foods. 2024 Jul 24;13(15):2325. doi: 10.3390/foods13152325 (PMC11311953; doi:10.3390/foods13152325)
Supplement: Supplementary file 1 [file foods-13-02325-s001.zip › foods-3097981-supplementary.pdf]

Table S1. Detailed summary of the available literature regarding the modification of starches, flours, and grains by ultrasound treatments.

Botanical origin / Reference	Treatment parameters / Method of analysis	Effect of treatment
**STARCHES**		
Potato starch<br><br>(Gallant et al., 1972)	• US bath; Freq.: 960 kHz and 280 kHz; Int.: ~15 W/cm^2; Time: 3, 6, 16 and 32 min; Temp.: 5 °C; Atm.: Air, O_2, H_2, CO_2, vacuum; Conc.: 3, 11 mg/mL; Vol.: 1, 2 mL<br>• Light microscopy (LM); Scanning electron microscopy (SEM)	In atmosphere of H_2, many deep pits were produced. In air or CO_2, pitting was less pronounced, but injury to other parts of the surface was greater. Virtually no effect was produced in vacuum and under CO_2. Damage increased with time and decreased with increasing starch concentration.
Potato starch<br><br>(Degrois et al., 1974)	• Freq.: 960 and 280 kHz; Int.: ~15 W/cm^2; Time: 3-32 min; Temp.: 5 °C; Atm.: Air, O_2, H_2, CO_2, vacuum; Conc.: 3, 11 mg/mL; Vol.: 1, 3 mL<br>• SEM, Optical microscopy	The type of gas affected the nature and degree of granule erosion. The damage caused to starch was greater at lower concentrations and volumes.
Potato starch<br>Sweet potato powder<br>(Azhar & Hamdy, 1979)	• US probe (1.9 cm tip Φ); Freq.: 20 kHz; Power: 300 W; Amp.: 98 %; Time: 15, 30 and 60 min; Temp.: 1 – 2 °C; Conc.: 1, 0.75 and 0.5 %<br>• Presence of reducing sugars; Hydrolysis of starch by β-amylase; Polyacrylamide-gel electrophoresis	A rapid decrease in relative viscosity of sonicated starch was determined, particularly during the first 5 min exposure. Maximum degradation of potato starch was observed after 30 min sonication, evidenced by the decrease in relative viscosity.
Waxy rice starch<br><br>(Isono et al., 1994)	• US probe (36 mm tip Φ); Freq.: 20 kHz; Power: 600 W; Time: 1, 2, 4, 8, 24 and 48 h; Temp.: 30, 40, 50, and 60 ºC.<br>• Gel permeation chromatography (GPC)	US decreased average molecular weight, accelerated by higher power. Number average molecular weight tended to a constant value at long sonication, and molecular weight distribution tended to be fairly narrow.
Wheat starch<br><br>(Seguchi et al., 1994)	• US probe (1/4 in tip Φ); Time: 300, 600, 900, 1200, 1500, 1800, 2100, 2400 and 2700 s; Temp.: 40 °C; Solv.: 90 % DMSO; Sample: 250 mg<br>• Gel filtration chromatography (molecular weight); Light scattering microscopy; Transmission electron microscopy (TEM)	With increasing US time, the solution turned more transparent, its viscosity decreased, and the linear increase of starch reducing power was observed. Wheat starch agglomerates were gradually reduced in size by shear force of sonication
Mung bean starch<br>Potato starch<br>Rice starch<br>(Chung et al., 2002)	• US probe (3.2 mm tip Φ); Power: 30 W; Time: 1, 3 and 5 min; Temp.: pre-heating at 95 °C; Conc.: 5 % (w/w). Sample: 28 g<br>• Apparent viscosity; Inherent viscosity; Paste clarity; Degree of polymerization (DP); Swelling power (SP)	Decrease of the apparent and inherent viscosities of starches after ultrasonication. Average degree of polymerization did not change by US. Starch paste became more transparent.
Corn starch<br>(Czechowska-Biskup et al., 2005)	• US bath; Freq.: 360 kHz; Power: 170 W/kg; Temp.: 22±2 °C; Atm: Ar, He and O_2; Sample: 10 mL<br>• Viscosity measurements (viscosity-average molecular weight.)	Starch molecular weight was reduced by US treatment. The yield of this degradation process depended on polymer concentration, ultrasound power and gas used to saturate the solution.
Corn starch<br><br>(Huang et al., 2007)	• US probe (6 mm tip Φ); Power: 500 W; Time: 3 - 15 min; Pulse: 15 s on / 5 s off; Conc.: 30 % (w/v); Sample: 200 g<br>• Degree of hydrolysis; Polarized light microscopy (PLM); SEM; X-ray diffraction (XRD); Differential scanning calorimetry (DSC); Reaction with propylene oxide; Pasting properties	The crystalline structure of treated corn starch did not change, but the amorphous area was slightly destroyed. Pores and channels were detected. Gelatinization enthalpy and temperature first increased and then slightly dropped with increasing degree of hydrolysis.
Starches: waxy maize, potato, tapioca, sweet potato, and corn<br>*(Sonication applied to gelatinized starches)*<br>(Iida et al., 2008)	• US [probe (12.7 mm tip Φ); Power: 120 W; Sample: 100g] [bath; Power: 100 W; Sample: 500 g]; Time: 30 min; Temp.: 60 °C; Conc.: 5, 10 %<br>• Degree of granule disintegration; Degree of solubilization; High performance gel permeation chromatography (HP-GPC); ^{1}H and ^{13}C Nuclear magnetic resonance (NMR) spectroscopy	Sonication drastically depressed the solution viscosity without altering the chemical structure of the polymer chains.<br>The molecular weight sharply decreased in the initial period of 10-30 min and thereafter the depolymerization proceed slowly.

Starches: normal maize, waxy maize, and amylomaize V<br>(Luo et al., 2008)	• US bath; Power: 100 W; Time: 30 min; Temp.: 30 °C; Conc.: 30 %; Sample: 100 g<br>• SEM; Laser light scattering (particle size); SP and solubility (S); XRD; DSC; Freeze-thaw stability (FTS); Pasting properties	Porous and a fissure were observed on the surface of treated starches. Swelling power, solubility and gelatinization transition temperatures were increased by US, and a viscosity drop was observed. US degraded preferentially the amorphous regions of starches.
Waxy rice starch<br><br>(Zuo et al., 2009)	• US bath; Freq.: 211 kHz; Power: 2.5, 4.1 W; Int.: 0.11, 0.18 W/cm^2; Time: ≤ 60 min; Temp.: 25–70 °C; Conc.: 5 % (w/w); Sample: 60 g<br>• Pasting properties; Particle size distribution (PSD); SEM; High-performance size exclusion chromatography-multiple laser light scattering (SEC-MALS)	A viscosity reduction was found in samples sonicated at high temperature. Particle size was reduced by US, while no surface damage was seen by SEM, and no reduction of starch molecules was found with SEC-MALS.
Starches: corn, potato, mung bean and sago<br><br>(Chan et al., 2010)	• US bath; Time: 10 min; Solv.: water and sodium dodecyl sulphate (2 % w/v); Conc.: 40 % (w/v); Sample: 250 g<br>• Amylose content; SEM; SP and S; Pasting properties [Rapid Visco Analyzer (RVA)]	Sonication appeared to induce a rough surface and fine fissures on starch granules. The combination of SDS and sonication increased amylose content, swelling and solubility of starches. Peak viscosity was increased while pasting temperature was reduced.
Corn starch<br><br>(Herceg et al., 2010)	• US [probe (7 mm tip Φ); Freq.: 24 kHz; Power: 100, 300, 400 W; Int.: 34, 55, 73 W/cm^2; Amp.: 100 %] [bath; Freq.: 24 kHz; Power: 300 W; Int.: 2 W/cm^2]; Time: 15 & 30 min; Conc.: 10 % (w/w); Vol.: 500 mL<br>• Pasting properties; S; Gel texture properties (GTP); Micrograph pictures; DSC	Sonication with probes caused lowering of T_O. US treatment caused disruption of starch granules and made them more permeable to water, resulting in increased solubility. More mechanical damage was observed when applying more powerful ultrasound.
Corn starch<br><br>(Jambrak et al., 2010)	• US [probe (7 mm tip Φ); Freq.: 24 kHz; Power: 100, 300, 400 W; Int.: 34, 55, 73 W/cm^2; Amp.: 100 %] [bath; Freq.: 24 kHz; Power: 300 W; Int.: 2 W/cm^2]; Time: 15, 30 min; Conc.: 10 % (w/w); Vol.: 500 mL<br>• DSC; Rheological properties; Turbidity; SP; Micrography	Ultrasonication distorted the crystalline region in starch granules, caused a decreased in gelatinization enthalpy, a significant decrease in consistency coefficient (*k*), and increase in swelling power. Micrography showed impact on the granules structure and size.
High-amylose maize starch<br>(Lima & Andrade, 2010)	• Melt-processing + Ultrasound; US probe (13 mm tip Φ); Freq.: 20 kHz; Power: 750 W; Amp.: 40 %; Time: 30 min; Temp.: 10 °C; Conc.: 5 g/L<br>• ^{1}H NMR; Viscosity measurements; XRD	US caused a significant reduction in intrinsic viscosity for the sample previously processed with the highest glycerol content, revealing a significant reduction in amylose molar mass.
Tapioca starch<br><br>(Manchun et al., 2012)	• US probe; Freq.: 24 kHz; Power: 400 W; Amp.: 50 and 100 %; Time: 10, 20 and 30 min; Conc.: 3 % (w/w)<br>• SEM; SP and S; XRD	US distorted the crystalline region in starch granules, especially at higher amplitude or longer time. Swelling power and solubility increased after treatments, associated with improved water absorption capacity.
Potato starch<br><br>(Zhu et al., 2012)	• US probe (13 mm tip Φ); Freq.: 20 kHz; Power: 60, 105, and 155 W; Time: 30 min; Pulse: 2 s on / 2 s off; Temp.: Controlled; Conc.: 10 % (w/w); Vol.: 30 mL<br>• SEM; PLM; XRD; Small angle X-ray scattering (SAXS)	US induced notch and groove on starch granule surface. B-type crystal structure was scarcely affected. Ultrasonication affected cluster structure, especially the crystalline region, with a reduction in the molecular order in crystalline lamellae. No particle size reduction was determined.
Potato starch<br>(Zuo et al., 2012)	• US probe (13 mm tip Φ); Freq.: 20 kHz; Power: 0 - 60 W; Time: 30 min; Temp.: 5 °C; Conc.: 0.1 % (w/w); Vol.: 5 mL<br>• LM; Starch granule damage; Rheological measurements	US caused starch surface damage. The number of defects first increased linearly with an increase in US power up to a threshold level. There was a linear dependence of the number of defects on the US power.
Waxy maize starch<br>Standard maize starch<br><br>(Bel Haaj et al., 2013)	• US probe (13 mm tip Φ); Freq.: 24 kHz; Power: 170 W; Time: > 75 min; Temp.: 8 °C; Conc.: 1.5 % (w/w); Vol.: 100 mL<br>• Wide-angle-X-ray diffraction (WAXD); Raman spectroscopy; Transmittance; PSD; Dynamic light scattering (DLS) (Z-average size); Field effect scanning electron microscopy (FE-SEM)	Particle size of starch granules decreased continuously with ultrasonication time, generating nanoparticles between 30 and 100 nm. Sonication seriously disrupted the crystalline structure of clustered amylopectin.

Starches: potato, wheat, corn, and rice<br><br>(Sujka & Jamroz, 2013)	• US probe; Freq.: 20 kHz; Power: 170 W; Time: 30 min; Temp.: 20 °C; Solv.: water and ethanol; Conc.: 30 % (w/v)<br>• Blue value and iodine absorption spectra; Fat and water absorption; Transmittance; Paste viscosity; Least gelling concentration; SP and S; SEM; TEM	Depolymerization of starch was higher when it was sonicated in water than in ethanol. Cracks and depressions were found on the surface of granules, especially in potato and wheat starches. Ultrasonication increased fat and water absorption, least gelling concentration, solubility and swelling power, and decreased starch paste viscosity.
Sweet potato starch<br><br>(Zheng et al., 2013)	• Freq.: single (25 and 80 kHz) and dual (25+80 kHz); Power: 720 W; Time: 10, 20, 30, 45 and 60 min; Temp.: 30±2 °C; Conc.: 5% (w/w)<br>• SEM; Starch-iodine complex absorption; Fourier transform infrared spectroscopy (FTIR); Pasting properties; S; Transparency	Starch-iodine complex analysis showed that US destroyed amylopectin and starch chains. FTIR showed damage to the crystalline structure. Peak viscosity was reduced, while solubility and transmittance were increased. Dual frequency US caused more changes than single frequency.
Non-waxy rice starch<br><br>(Yu et al., 2013)	• US probe (6 and 10 mm tip Φ); Freq.: 24 kHz; Power: 100, 500 and 1000 W; Time: 0 - 120 min; Conc.: 5 % (w/v); Sample: 15 g; Vol.: 300 mL<br>• DSC	Higher temperatures were reached in starch suspensions when sonicating at higher power. High ultrasound power and strong intensity can effectively change the gelatinization and retrogradation properties or rice starch.
Pinhão starch<br><br>(Gonçalves et al., 2014)	• US probe; Power: 100 W; Time: 30 cycles of 1 min on / 1 min off; Temp.: controlled; Sample: 10 g; Vol.: 500 mL<br>• PSD; Total starch; Amylose content; Color; XRD; SEM	US-modified starch achieved nanometric size, with mean particle size of about 453 nm. Amylose content was not modified by US treatment.
Corn starch<br><br>(Hu et al., 2014)	• US bath; Freq.: single (20 and 25 kHz) and dual (20+25 kHz); Time: 5, 10, 15, 20, 30, 40 min; Temp.: 30 °C; Solv.: ethanol solution (20 % v/v); Conc.: 5 % (w/v); Sample: 50 g.<br>• Transmittance; GTP; DSC; FTS; XRD	Transparency of starch paste was improved by US, while hardness, brittleness, elasticity, adhesiveness, conglutination degree, chewiness, recoverability, crystallinity and *ΔH* decreased. Dual frequency treatment was found to be more effective than single frequency treatment.
Taro starch<br><br>(Sit et al., 2014)	• US probe (7 mm tip Φ); Freq.: 30 kHz; Power: 100 W; Amp.: 50 and 100 %; Time: 5, 10 min; Cycle: 0.5, 1; Temp.: 20 °C; Sample: 100 g<br>• SP and S; Clarity of starch paste; Color; Pasting properties; Texture analysis; FTS	A significant increase in swelling, solubility, pasting and texture properties of the ultrasonically extracted starch was observed. The freeze-thaw stability was slightly better after ultrasonication. The whiteness of the starch powders was lower after ultrasonication.
Corn starch<br><br>(Amini et al., 2015)	• US probe (3 mm tip Φ); Freq.: 24 kHz; Power: 150 W; Amp.: 0, 50, 100 %; Cycle: 80 %; Time: 5, 10, 15 min; Temp.: 25 - 65 °C; Conc.: 10, 15, 20 % (w/w); Vol.: 50 mL<br>• LM; SEM; SP and S; Transmittance; DP; DSC; XRD; Rheological properties	The influence of sonication strongly depended on temperature and treatment time, while concentration and amplitude had little influence on functional and rheological properties. The most effective parameter was sonication temperature followed by exposure time and starch concentration.
Corn starch<br><br>(Hu et al., 2015)	• US bath; Freq.: single (20 and 25 kHz) and dual (20+25 kHz); Time: 40 min; Temp.: 30 °C; Conc.: 5 % (w/w)<br>• Pasting properties; SEM; Determination of cavitation yield by iodine release method	Starch granules presented many dents and holes after treatments. Gel properties were decreased, while thermal stability and retrogradation was enhanced. Peak viscosity decreased as US frequency increased. Dual-frequency treatment caused more obvious damage than single-frequency.
Banana starch<br>(Orsuwan & Sothornvit, 2015)	• US probe (6 mm tip Φ); Freq.: 20 kHz; Power: 130 W; Amp.: 40, 60 and 80 %; Time: 30 and 60 min<br>• SEM; FTIR; SP and S; DSC	US treatment of miniemulsion cross-linked banana starch showed a lower range of gelatinization temperature than untreated. SEM showed that US caused slight fragmentation and formation of a rough surface.
Pinhão starch<br><br>(Pinto et al., 2015)	• US probe (5 mm tip Φ); Single US treatment, dual ANN+US, HMT+US, US+ANN and US+HMT treatments; Freq.: 20 kHz; Amp.: 50 %; Time: 90 min; Pulse: 30 s on / 5 s off; Temp.: controlled; Conc.: 25 % (w/v); Sample: 100 g; Vol.: 400 mL<br>• HP-GPC; WAXD; SEM; SP and S; RVA; DSC	Relative crystallinity decreased in sonicated starch, where no visible cracks were detected. Sonication provided high swelling power, with no effect on solubility. When US was applied as second treatment, an peak viscosity increase was determined. US-treated starch presented the highest breakdown viscosity. Gelatinization enthalpy decreased after US.

Plantain starch<br>Taro starch<br>(Carmona-García et al., 2016)	• US probe (7 mm tip Φ); Freq.: 25 kHz; Power: 80 W; Amp.: 20 %; Time: 20 and 50 min; Temp.: 4 °C; Conc.: 5 % (w/v); Vol.: 100 mL<br>• Optical microscopy; SEM; Laser diffraction analysis; SP and S; XRD; DSC; Pasting properties; Rheological properties	Ultrasounds caused profound cavities and fractures, without causing a reduction of granule size. Peak viscosity, swelling power and solubility increased after treatments. Sonication of high granule size starch resulted in more pronounced decrease in storage modulus (*G′*).
Corn starch<br>*(Sonication applied to gelatinized starches)*<br>(Kang et al., 2016)	• US probe (13 mm tip Φ); Freq.: 20 kHz; Power: 13.5 and 29.9 W; Time: ≤ 30 min; Conc.: 5 and 10 % (w/w); Vol.: 20 mL<br>• LM; FTIR; Rheological properties; Particle size measurements (dynamic light scattering)	Viscosity and hydrodynamic radius decreased with increasing US time. FTIR showed molecular scission at C-O-C bond of α-1,6 glycosidic linkage, with the extent of breakage being inversely correlated to amylose content. High-amylose starch pastes were more resistant to US.
Normal potato starch<br>Waxy potato starch<br><br>(Bai et al., 2017)	• US bath; Freq.: 1 MHz, 850 kHz and 500 kHz; Power: 0.2, 2 and 3.7 W; Time: 2.5, 5, 10, 15, 20, 25, 30, 45, 60, 90, 120 min; Temp.: 2 °C; Conc.: 1 % (w/w); Sample: 10 g<br>• LM; SEM; FTIR; PSD	The number of pits per starch granule was independent of the amylose content, but strongly depended on granule size. Small granules were more pitted than the large ones. High frequency ultrasound was more favorable to produce ultrasonic pitting.
Corn starch<br><br>(Flores-Silva et al., 2017)	• US probe (7 mm tip Φ); Freq.: 24 kHz; Int.: 300 W/cm^2; Amp.: 80 %; Time: 1, 2, 4, 8 and 16 min; Temp.: 20 °C; Conc.: 30 % (w/v)<br>• Laser diffraction analysis; SEM; XRD; FTIR ; DSC; Apparent viscosity; *In vitro* starch digestibility (IVSD)	SEM images showed disruption of granules. XRD indicated increased relative crystallinity content. Resistant starch content increased after 16 min sonication, attributed to morphological and crystallinity changes that reduced the structure of the starch granule channels.
Starches: Potato, wheat, corn, and rice<br>(Sujka, 2017)	• US probe; Freq.: 20 kHz; Power: 170 W; Time: 30 min; Temp.: 20 °C; Solv.: water and ethanol; Conc.: 30 % (w/v)<br>• Low temperature nitrogen adsorption (to investigate porosity of starch)	Modification of starch with US resulted in the formation of new pores in the studied range of diameter. Results varied due to botanical origin of starch and solvent used.
Yam starch<br><br>(Bernardo et al., 2018)	• US probe; Freq.: 25 kHz; Power: 450 W; Amp.: 12, 40, 68 and 70 %; Time: 3, 6, 9 and 15 min; Conc.: 50 % (w/w)<br>• Color; SEM; LM; PSD; XRD; SP and S; Paste clarity; RVA; DSC; Absolut density	Starch extraction yield increased with ultrasonication. Starch surface became damaged, and amorphous region was reduced, but no change was observed in the crystalline pattern. Except for starches treated at 70 % amplitude for 15 min, all others showed no change of peak viscosity.
Maize starch<br><br>(Flores-Silva et al., 2018)	• US probe (7 mm tip Φ); Dual US-HMT and HMT-US treatments; Freq.: 50 kHz; Int.: 300 W/cm^2; Amp.: 80 %; Time: 1, 2, 4, 8 and 16 min; Temp.: 20 °C; Conc.: 30 % (w/v)<br>• FTIR; DSC; IVSD	Dual treatments rearranged starch structure, increasing RS content. HMT-US produced thermos-stable SDS and RS. FTIR indicated that changes in SDS and RS fractions may be associated with variations in the packing of double helices within the crystalline lamella.
Corn starch<br><br>(Li et al., 2018)	• US bath; Freq.: 40 kHz; Power: 420, 480 and 540 W; Time: 20, 30 and 40 min; Temp.:40, 50 and 60 °C; Conc.: 30 % (w/v)<br>• Hydrolysis degree; RVA; DSC; SEM; PLM; FTIR; XRD; HP-GPC	US reduced liquefaction process time and resulted in increased hydrolysis degree. The appearance of notch and groove on granules surface was detected, while polarized cross became smaller or even disappeared.
Cassava starch<br><br>(Monroy et al., 2018)	• US probe (13 mm tip Φ); Power: 750 W; Amp.: 40 and 60 %; Time: 5, 10, 20 min; Temp.: with and without control; Conc.: 5 % (w/v)<br>• SEM; Confocal laser scanning microscopy (CLSM); Granule size distribution; Attenuated total reflection FTIR (ATR-FTIR); XRD; DSC; SP; Rheological characterization	US produced morphological and crystallinity changes, reduction of particles size and increase of swelling power. Gelatinization enthalpy was decreased, while transition temperatures were not affected. Higher *G′* values were observed for the treatment performed for 20 min at 60% amplitude.
Foxtail millet starch<br><br>(Babu et al., 2019)	• US bath; Single US treatment and dual ANN+US and US+ANN treatments; Freq.: 33 kHz; Time: 30 min; Temp.: 50 °C; Conc.: 50 % (w/v); Sample: 200 g; Vol.: 400 mL<br>• Amylose content; Water absorption capacity (WAC); SP; Acid resistance; Shear and Freeze-thaw stability; IVSD; RVA; Color; GTP; Gel filtration	Modified starches contained higher amylose. US had a predominant effect on RS level. Pasting properties were increased by sonication, while color was not affected. Hardness of gels was decreased by treatments. Ultrasonication led to increased proportion of very short and short chains after depolymerization of long chain amylose and amylopectin molecules.

	chromatography and number average molecular weight; XRD; FTIR; SEM	FTIR indicated reduced values of the 1047/1022 cm^{-1} ratio. SEM displayed deep pitting and cracks on granular surface after US.
Retrograded starch (RS3)<br><br>(Ding et al., 2019)	• US probe (6 mm tip Φ); Freq.: 20 kHz; Power: 100, 200; 300, 400, 500 and 600 W; Time: 30 min; Pulse: 2 s on / 2 s off; Temp.: controlled; Conc.: 10 % (w/v)<br>• SEM; PSD; Zeta potential; Apparent amylose content (AAC); XRD; FTIR; DSC; IVSD; SP and S	High power resulted in more compact block-shape structure. US decreased the long-range orders but increased the median particle size, short-range orders, and V-type polymorph of RS3. Some RDS fractions were converted into SDS and/or RS. The degree of change in molecular structures and starch digestibility depended on the applied US power.
Oat starch<br><br>(Falsafi et al., 2019)	• US [probe (12 mm tip Φ); Freq.: 20 kHz; Int.: 39, 48, 63 W/cm^2; Pulse: 5 s on / 5 s off] [bath; Power: 350 W; Int.: 5 W/cm^2]; Time: 10 and 20 min; Temp.: 25 °C; Conc.: 5 %; Vol.: 100 mL<br>• SEM; Granule size; AAC; DSC; XRD; Paste viscosity; SP and S; Transmittance; Oil and water absorption; Gel hardness; Syneresis	Fissures and pores were detected on the surface of granules treated with the probe. Size reduction was observed at the highest intensity. US increased amylose content, swelling power, solubility, transmittance, water and lipid holding capacity, and gel hardness was diminished. Gelatinization temperatures increased and crystallinity degree decreased.
Potato starch<br>Millet starch<br>(Hu et al., 2019)	• US bath; Freq.: single (40 kHz) and dual (40+80 kHz); Power: ≤ 720 W; Temp.: 25 and 60 °C; Conc.: 5 % (w/w)<br>• Pasting properties; PSD; SP; FTIR; DSC; XRD; SEM	US caused the partial collapse of potato starch. Reduction of particle size, peak viscosity and relative crystallinity was greater in potato starch. Dual frequency US caused more changes than single frequency.
Starches: Wheat, barley, rice, and maize<br><br>(Kaur & Gill, 2019)	• US probe (2 mm tip Φ); Freq.: 24 kHz; Power: 100 W; Amp.: 100 %; Time: 15 and 30 min; Temp.: controlled; Vol.: 50 mL<br>• SP and S; PSD; SEM; XRD; FTIR; Gel rheological properties; IVSD	US increased swelling power and solubility. Depressions and pores were seen on granules' surface. Sonicated starches presented increased RDS and RS content, while SDS content decreased. Increase G' and G" were determined after treatments of 15 min, and decreased values after 30 min.
Millet starch<br><br>(Li et al., 2019)	• US bath; Freq.: 25 + 40 + 80 kHz; Power: 700 W; Time: 15, 30, 45, 60 min; Conc.: 10, 15, 20, 25 and 30 % (w/w); Sample: 20 g<br>• Pasting properties; SP; Transparency; ATR-FTIR; DSC; XRD; *In vitro* enzymatic digestibility; SEM	US increased swelling power (at short times) and transparency. The 1047/1022 ratio was gradually increased with the increase of concentration and time. US increased ΔH and *in vitro* enzymatic digestibility. SEM showed cracks and pores, even some broken granules.
Cassava starch<br>Corn starch<br>Yam starch<br>(Minakawa et al., 2019)	• US probe (1.27 mm tip Φ); Freq.: 20 kHz; Time: 30 min; Temp.: 25 °C; Conc.: 10 % (w/w); Vol.: 100 mL<br>• SEM; Atomic force microscopy (AFM); Blue value (DP of amylose and amylopectin); XRD; FTRI; Thermogravimetric analysis (TGA)	Starch microparticles and nanoparticles were obtained after US. Yam starch, exhibiting higher amylose content, was more susceptible to ultrasonication, generating smaller starch nanoparticles and microparticles than those obtained from corn and cassava starches.
Potato starch<br>*(Sonication applied to gelatinized starches)*<br>(Nie et al., 2019)	• US probe (7 mm tip Φ); Freq.: 25 kHz; Power: 300 W; Time: 60 min; Temp.: 10 ºC; Conc.: 4, 5, 6, 7 and 8 % (w/v); Sample: 1 mL<br>• FTIR; properties of retrogradation; XRD; GTP; GPC; Intrinsic viscosity; free radical content and molecular weight distribution	A remarkable decrease in gel texture properties and intrinsic viscosity was obtained when sonication time was prolonged, the intensity was increased and when starch concentration was reduced. GPC showed that the average molecular weight decreased after ultrasonication.
Waxy corn starch<br><br>(Yang, Lu et al., 2019)	• US probe (38 mm tip Φ); Freq.: 15 kHz; Power: 100 and 400 W; Time: 40 min; Temp.: 25 °C; Conc.: 30 % (w/w)<br>• High-Performance Anion-Exchange chromatography (HPAEC-PAD); HPSEC-MALLS; ^1H NMR; ^{13}C CP/MAS NMR; XRD; SEM; PSD; Pasting properties; DSC	Lower proportion of linear chains and higher proportion of external chain were observed in US starch. Lower degree of branching was observed in US samples, and α-1,4 glycosidic linkages were more stable than α-1,6 glycosidic linkages. ^{13}C NMR indicated that double helices content was decreased, and single helix and amorphous components were increased.
Rice starch<br><br>(Yang, Kong et al., 2019)	• US probe (10 mm tip Φ); Freq.: 22 kHz; Power: 150, 300, 450 and 600 W; Time: 20 min; Pulse: 5 s on / 5 s off; Temp.: 25 °C; Conc.: 30 % (w/w); Vol.: 50 mL<br>• XRD; FTIR; Raman spectroscopy; HPAEC; SEM; PSD; DSC; RVA	US slightly destroyed the amorphous region of granules, while the crystalline pattern remained unchanged. US induced fissures and pores on granules surface. Peak and breakdown viscosities increased, while gelatinization enthalpy decreased.

Potato starch<br><br>(Cao & Gao, 2020)	• US bath; Single US treatment and dual US+electric field and electric field+US treatments; Freq.: 20 kHz; Power: 600 W; Time: 30 min; Temp.: 30 °C; Conc.: 20 % (w/w)<br>• SEM; XRD; FTIR; AAC; Granule size; Pasting behavior; Transmittance; WAC; SP; IVSD; Texture properties; DSC	Deep pitting was observed in treated granules. US treatment increased relative crystallinity, granule size and amylose content. Peak viscosity was increased, and setback reduced. RS and RDS contents were increased after US, while SDS was reduced. Hardness of US-gels was improved. T_o, T_p and T_c values were higher after ultrasonication.
Sweet potato starch<br><br>(Jin et al., 2020)	• Freq.: 20 kHz; Int.: 2, 4, 8, 12, 16 W/mL; Time: 10, 15, 20, 25, 30 min; Pulse: 3 s on / 5 s off; Temp.: 20, 30, 40, 50 and 60 °C; Conc.: 60, 80, 100, 125, 150 and 200 g/L<br>• RVA; DSC; FTIR; XRD; SEM	US decreased peak and setback viscosities, and the gelatinization range and enthalpy. FTIR indicated damage to the ordered structures and crystallization zone. Relative crystallinity was reduced by 15 %. US destroyed the surfaces and the linkages between starch granules.
Wheat starches<br><br>(Karwasra et al., 2020)	• US probe (2 mm tip Φ); Freq.: 30 kHz; Int.: 600 W/cm^2; Amp.: 100 %; Time: 15 and 30 min; Cycle: 80 %; Conc.: 10 % (w/v); Vol.: 25 mL<br>• SP and S; Oil absorption capacity (OAC); Amylose content; SEM; XRD; FTIR; PSD	Scraps and deformities were observed on granule surfaces after US, which were more prominent in large size granules. Relative crystallinity increased after US. FTIR spectra showed no new or broken bonds after US, whereas the absorbance ratio 1022/995 decreased significantly.
Purple taro starch<br><br>(Martins et al., 2020)	• US probe; Freq.: 20 kHz; Amp.: 40, 50 and 60 %; Time: 30 min; Temp: controlled; Conc.: 10 % (w/v)<br>• Color; Thermogravimetric analysis; DSC; XRD; RVA; Field emission gun-SEM	US treatments led to an increase in thermal stability, and to a reduction of the degree of relative crystallinity. The size of granules showed a slight decrease, with no signs of agglomerations.
Taro starch<br><br>(Thomaz et al., 2020)	• US probe; Dual HMT and US treatments; Freq.: 20 kHz; Amp.: 60 %; Time: 30 min; Conc.: 10 % (w/v)<br>• DSC, XRD; SEM; RVA	Morphology was not affected by US. Relative crystallinity increased, and RVA profiles decreased after ultrasonication. Dual treatment resulted in lower gelatinization temperature range and decreased enthalpy.
Chestnut starch<br><br>(Wang, Wu et al., 2020)	• US probe (6 mm tip Φ); Treatments: Single US and dual US+MW (UM) and MW+US (MU); Freq.: 20 kHz; Power: 500 W; Time: 60 min; Pulse 2 s on / 2 s off; Temp.: < 25 °C; Conc.: 10 % (w/v); Sample: 2 g<br>• SEM; PLM; XRD; FTIR; DSC; RVA; SP; FTS; WAC and OAC	UM and MU dually-modified samples exhibited more severe surface damage, lower crystallinity and lower ΔH than the native and single-treated starches. SP, peak, trough, final and breakdown viscosities, and pasting temperature all decreased by single US and dual modification.
Sweet potato starch<br><br>(Wang, Xu et al., 2020)	• US probe (13 mm tip Φ); Freq.: 20 kHz; Power: 300 W; Time: 15, 20, 25 and 30 min; Pulse: 3 s on / 5 s off; Temp.: 30 °C; Conc.: 6 % (w/w)<br>• Amylose content; SEM; PSD; XRD; FTIR; Raman spectroscopy; DSC; SP and S; RVA; Dynamic rheological properties	Pores and cracks were observed after US. Structural disorganizations were more evident with increasing time, especially in crystallinity, short-range molecular order and ordered molecular structures. US increased SP and S, decreased pasting properties, and strengthened gels.
Pea starch<br><br>(Han et al., 2021)	• US probe (6 mm tip Φ); Single US treatment and dual US+HMT and HMT+US treatments; Freq.: 20 kHz; Power: 300 W; Time: 20 min; Temp.: 0 °C; Conc.: 10 % (w/w); Vol.: 300 mL<br>• LM; SEM; CLSM; Amylose content; Molecular weight; Chain length distribution (HPAEC-PAD); XRD; FTIR; IVSD; S; SP; RVA	Pea starch maintained the original morphology and C-type crystallinity after US, but 4 h or more of HMT and HMT+US changed it from C-type to A-type. US decreased molecular weight and relative crystallinity, while increased amylose content, increased RDS and RS, and all viscosity parameters in RVA.
Corn starch<br>Potato starch<br>Pea starch<br><br>(Ouyang et al., 2021)	• US probe (6 mm tip Φ); Freq.: 25 kHz; Power: 300 W; Time: 30 min; Pulse: 5 s on / 5 s off; Temp.: 5, 15, 25, 35, 50 °C; Conc.: 30 % (w/w); Vol.: 100 mL<br>• AAC; SEM; FTIR; XRD; DSC; degree of gelatinization and retrogradation; IVSD	The degree of surface damaged varied depending on starch source. Short- and long-range orders were increased in corn starch, while decreased in potato and pea starches, by US treatments. AAC was increased for potato starch and decreased for corn and pea starches.
Corn starch<br>Cassava starch<br>(Rahaman et al., 2021)	• US bath; Freq.: 40 kHz; Amp.: 99 %; Time: 10 and 20 min; Temp.: 26±2 °C; Conc.: 20 %	Groove and notch appeared on the surface of the starch granules after US. T_O did not change with ultrasonication, but ΔH decreased. XRD showed slight decrease in the crystallinity degree after US.

	• XRD; FTIR; DSC; SEM	
Arrowhead starch<br><br>(Raza et al., 2021)	• US bath; Freq.: tri-frequency treatment (20/40/60 kHz); Power: 300, 600 and 900 W; Time: 15 and 30 min; Pulse: 10 s on / 4 s off; Temp.: 24 ºC; Conc.: 10 % (w/v)<br>• SP and S; Water and oil holding capacities; PSD; FTIR; XRD; Rheological properties; SEM; DSC	Treatment led to an increase in SP, S and water and oil holding capacities. Increase of frequency and power led to an increase in onset gelatinization temperatures. US caused cracks and roughness on starch granules in a power-dependent manner.
Waxy maize starch *(Sonication applied to gelatinized starches)*<br>(Wei et al., 2021)	• US probe (13 mm tip Φ); Freq.: 25 kHz; Power: 800 W; Time: 5, 10, 15, 30, 60 and 120 min; Temp.: 20 ºC; Conc.: 1 % (w/v); Vol.: 500 mL<br>• High-performance size exclusion chromatography (HPSEC); ^{1}H NMR	Weight-average molecular weight was decreased with extension of the processing time. Break points occurred at B3 and B2 chains with an increase of A chains contents, resulting in a much narrower molecular size distribution.
Corn starch<br>Potato starch<br>Pea starch<br>(Zhang et al., 2021)	• US probe (6 mm tip Φ); Freq.: 25 kHz; Power: 100, 200, 300, 400, 500 and 600 W; Time: 5, 10, 15, 20, 25, 30 min; Pulse: 5 s on / 5 s off; Temp.: 25 °C; Conc.: 30 % (w/w)<br>• AAC; FTIR; DSC; IVSD; Rheological properties	Power and time decreased AAC in corn and pea starches, while increased it in potato starch. US enhanced 1047/1022 values of corn starch, whereas those of potato and pea starches were decreased. Us decreased the RS content of pea and potato starches, but increased that corn starch.
Cowpea starch<br><br>(Acevedo et al., 2022)	• US probe (13 mm tip Φ); Dual HMT+US treatment; Freq.: 20 kHz; Amp.: 80 %; Time: 30 min; Pulse: 2 s on / 2 s off; Temp.: 25 – 30 °C; Conc.: 10 % (w/v); Sample: 100 mL<br>• SEM; ^{1}H NMR; XRD; DSC; IVSD; Pasting properties	Granule shape and XRD pattern were not modified by US. ^{1}H NMR revealed that US decreased amylopectin branching degree. Gelatinization enthalpy was not affected by treatment. SDS was increased up to 30 % by HMT-US treatments. Pasting viscosity of starch was decreased by US.
Rice starch<br><br>(Li et al., 2022)	• Single US treatment and dual Vacuum+US treatments; Power: 300 W; Time: 15 and 30 min; Conc.: 25 % (w/w)<br>• SEM; PSD; SAXS; XRD; FTIR; Starch chain length distribution (using ion chromatography system); DSC; Pasting properties	Vacuum combined with US treatment could result in severe shrinkage and damage of starch granules. Treatment reduced average particle size with narrowed distribution range, decreased ordering degree of lamellar architecture and shortened chain length of rice starch. Modified starch presented reduced relative crystallinity, and gelatinization enthalpy.
Banana starch<br><br>(Sun et al., 2022)	• US treatments and resveratrol complexation; Freq.: 20 kHz; Amp.: 40, 60, 80 and 100 %; Time: 10 min; Temp.: controlled; Conc.: 1.0 % (w/v)<br>• SP and S; OAC; FTS; DSC; Rheological properties; IVSD; FTIR; XRD; SEM	Solubility, thermal stability, the storage and loss moduli, and digestion resistance were improved by US treatment. XRD and FTIR spectroscopy demonstrated that complex structures became more compact and organized, whereas crystalline patterns were unchanged.
Kiwi starch<br><br>(Wang, Lv et al., 2022)	• US probe (10 mm tip Φ); Freq.: 20-25 kHz; Power: 200, 400 and 600 W; Time: 10, 20 and 30 min; Temp.: controlled; Conc.: 5 % (w/v)<br>• SEM; PLM; PSD; XRD; FTIR; SP; WSI; OAC; DSC; RVA; GTP; Rheological properties; starch content; AAC; IVSD	US formed holes and cracks on the surface, and reduced the particle size and the short-range molecular order of kiwi starch, while increasing AAC, SP, WSI, and viscosity. US reduced the content of RDS and SDS, and enhanced the content of RS.
Normal maize starch<br>Potato starch<br><br>(Wang, Wang et al., 2022)	• US probe (6 mm tip Φ); Single US treatment and dual US+MW and MW+US treatments; Freq.: 20 kHz; Power: 12.4 W; Int.: 43.9 W/cm^{2}; Time: 60 min; Pulse: 2 s on / 2 s off; Temp.: controlled; Conc.: 10 % (w/v); Sample: 2.0 g<br>• Total starch (TS); Damaged starch; SEM; PLM; PSD; XRD; DSC	Ultrasonication loosened the internal space and destroyed the structure of starch granules, increased starch damage, decreased relative crystallinity and increased the median size values. The type of starch influenced the effect of US treatment.
White finger millet starch<br><br>(Amarnath et al., 2023)	• US bath; Single US treatment and dual US+ANN and ANN+US treatments; Freq.: 33 kHz; Time: 30 min; Temp.: 50 ºC; Ratio: 1:2 (starch:water)<br>• AAC; Color; WAC; SP; S; FTS; DSC; GTP; XRD; FTIR; SEM	WAC and SP were increased by US. Dual US+ANN treatment increased amylose content (31-33%), and *ΔH*. US led to cracks on starch granules surface.

Pea starch<br><br>(Han et al., 2023)	• US probe (6 mm tip Φ); Freq.: 20 kHz; Power: 300 W; Time: 20 min; Temp.: 0, 25, 35, 45 ºC; Sample: 10 g; Vol.: 100 mL<br>• SEM; CLSM; Amylose content; Molecular weight; Chain length distribution (HPAEC-PAD); XRD; FTIR; Raman spectroscopy; SAXS	US caused a pitted surface and endowed a looser structure and higher enzyme susceptibility as the temperature increased above 35 ºC. US reduced short-range order and increased the thickness of semi-crystalline and amorphous lamellae by inducing starch chain depolymerization.
Pea starch<br><br>(Hu et al., 2023)	• US probe (6 mm tip Φ); Single US treatment and dual treatments with bamboo leaf flavonoid; Power: 400 W; Time: 20 min; Pulse: 3 s on / 5 s off; Temp.: 25 ºC; Conc.: 15 % (w/w); Vol.: 100 mL<br>• Rheological properties; DSC; PSD; SEM; SAXS; XRD; FTIR; ^{1}H NMR; IVSD	Morphology and crystal structure of granules were destroyed by US, promoting starch retrogradation and digestion. Ultrasonication improved gel viscosity and elasticity but decreased pasting enthalpy.
Arrowhead starch<br><br>(Raza et al., 2023)	• US bath; Freq.: Dual (20/40 kHz); Power: 300 W/L; Time: 20 min; Pulse: 10 s on / 4 s off; Sample: 10 g.<br>• XRD; FTIR; SEM; DSC; PSD; IVSD	The heat-US treatment increased the dispersion of phenolic acids (gallic acid and ferulic acid) in the amylose cavity. Cracks, fissures, and ruptures were observed in treated starch-phenolic acid complexes. Treatment improved digestion resistance of the complexes.
Sweet potato starch<br><br>(Ulfa et al., 2023)	• US bath; Freq.: 40 kHz; Time: 5-15 min; Temp.: 50-60 ºC; Conc.: 1 % (w/v); Sample: 100 g; Vol.: 100 mL<br>• S and SP; Water binding capacity; TS; Amylose content; Color; SEM; RVA; FTIR	Using 59.58 ºC for 8.01 min gives the best products on US-modified starch. A rougher surface and pores were detected after US treatments.
Quinoa starch<br>Maize starch<br><br>(Wei et al., 2023)	• US probe (1/2 " tip Φ); Freq.: 20 kHz; Amp.: 50 %; Time: 2, 4, 8, 14 and 22 h; Pulse: 3 s on / 1 s off; Temp.: < 25 ºC; Conc.: 7.5 % (w/w); Vol.: 200 mL.<br>• SEM; PSD; XRD; DSC; SP; WSI; AAC; Pasting properties; GTP; Dynamic oscillation tests; HPSEC	Short term US caused an increase in swelling power in quinoa starch. Increasing US times led to degradation of amylopectin external chains, as seen by a decrease of relative crystallinity by x-ray diffraction. The increase of AAM after ultrasonication resulted in increased gel hardness.
Corn Starch<br>(Yilmaz & Tugrul, 2023)	• US probe; Freq.: 20 kHz; Dual US+MW and MW+US treatments; Time: 20, 30 and 40 min; Temp.: 35 ºC; Conc.: 30 % (w/v)<br>• SEM; FTIR	The functional groups of natural corn starch were not changed under these treatments. Starches completely lost their original structure after treatment, showing an irregular surface with grooves and notches.
Normal maize starch<br>Potato starch<br><br>(Zhou et al., 2023)	• US probe; Single US treatment and dual US+MW and MW+US treatments; Freq.: 20 kHz; Power: 500 W; Time: 60 min; Pulse: 2 s on / 2 s off; Temp.: < 25 ºC; Conc.: 10 % (w/v); Sample: 2.0 g<br>• S and SP; WAC and OAC; FTS; IVSD	The solubility, water and oil absorption capacity, and the freeze–thaw stability of both starches increased after treatment. The ultrasound treatment and dual treatment increased the starch digestibility, probably due to the loosening of the internal structure of starch by ultrasound.
Purple rice starch<br><br>(Su et al., 2024)	• US probe (20 mm tip Φ); Single US treatment and dual HMT+US treatment; Freq.: 50 Hz; Power: 300, 600, 900 W; Time: 30 min; Pulse: 1 s on / 1 s off; Temp.: Controlled; Conc.: 5 % (w/w); Vol.: 100 mL<br>• Amylose content; SEM; PSD; DSC; Rheological properties; FTIR; XRD; GTP	US induced alterations in the granule surface and internal structure of the starch, while having a minimum impact on the overall integrity of the granules. Rheological properties showed a synergistic effect of HMT with US, enhancing the fluidity of purple rice starch and its resistance to deformation.
**FLOURS**		
Quinoa flour<br><br>(Zhu & Li, 2019)	• US probe (3/8" tip Φ); Freq.: 20 kHz; Power: 250 W; Pause: 80 %; Time: 1.2, 2.4, 4.8, 9.6 and 19.2 h; Temp. < 25 °C; Conc.: 5 % (w/v); Sample: 10 g<br>• SP; Water solubility index (WSI); Pasting properties; GTP; DSC; IVSD; Total phenolic content; *In vitro* antioxidant activity	Treatment for more than 5 h significantly increased water solubility and *in vitro* starch digestibility of quinoa flour, while decreasing gelatinization temperatures and enthalpy, viscosity during pasting event, gelling capacity, *in vitro* antioxidant activity, and total phenolic content. Changes depended

		mostly on treatment time, and indicated degradation and modifications of the chemical components of quinoa flour.
Flours: Purple dawn sweet potato, red sweet potato, and wheat<br><br>(Cui & Zhu, 2020)	• US probe (13 mm tip Φ); Freq.: 20 kHz; Power: 750 W; Amp.: 50 %; Time: 2, 4, 8, 16 and 20 h; Pulse: 3 s on / 1 s off; Temp.: 20±0.5 °C; Conc.: 10 % (w/v); Sample: 20 g<br>• SEM; PSD; FTIR; DSC; α-amylase activity; Pasting properties; GTP; IVSD; Color; Total phenolic content; *In vitro* antioxidant activity	US caused erosion on starch granules surface, reduced the particle size and starch crystallinity. Color changes were caused by prolonged ultrasound processing. Longer treatments decreased gelatinization enthalpy, pasting viscosities, gelling capacity, while increased in vitro starch digestibility of flours. Longer US times reduced total phenolic contents and *in vitro* antioxidant activities of sweet potato flour.
Rice flour<br><br>(Vela, Villanueva, Solaesa, et al., 2021)	• US probe (22 mm tip Φ); Freq.: 24 kHz; Power: 180 W; Time: 2, 5, 10, 20, 40 and 60 min; Cycle: 80 %; Temp.: 20 °C; Conc.: 5, 10, 20 and 30 % (w/w); Sample: 400 g<br>• PSD; SEM; Starch damage; Hydration properties; XRD; FTIR; DSC; Pasting properties; Rheological properties	Particle size was reduced by US, which improved the water absorption ability. US-flours showed reduced ΔH and narrowing of ΔT. XRD patterns were affected by US, as well as amide I secondary structures. Pasting profiles were reduced with increasing time and was not influenced by concentration. US led to gels with higher strength.
Rice flour<br><br>(Vela, Villanueva, & Ronda, 2021)	• US probe (22 mm tip Φ); Freq.: 24 kHz; Power: 180 W; Time: 60 min; Cycle: 80 %; Temp.: 20, 40, 50 and 60 °C; Conc.: 10 % (w/w); Sample: 400 g<br>• PSD; SEM; Starch damage; Hydration properties; XRD; FTIR; DSC; Pasting properties; Rheological properties	Starch long range crystallinity order and protein secondary structure was affected by US. A narrowing of ΔT was determined by higher treatment temperature. Pasting viscosities were significantly decreased, and rheology indicated reduction of the elastic and viscous moduli and the loss tangent after treatments.
Potato flour<br><br>(Hou et al., 2023)	• US probe (6 mm tip Φ); Freq: 25 kHz; Power: 200, 300, 400, 500 and 600 W; Time: 20, 40, 60, 80 and 100 min; Cycle: 5 s on / 5 s off; Temp.: 25 ºC; Conc.: 35 % (w/v)<br>• Blue value; Oil holding capacity; SP and S; FTS; Transparency; IVSD; DSC; SEM; FTIR; XRD	Sonicated flour exhibited an increase in SP, S, syneresis rate and transparency, and a decrease of ΔH. US decreased the RDS content, while increased the RS and SDS contents. US increased particle size, and the crystallinity of the sonicated flour.
White tef flour<br>Brown tef flour<br><br>(Vela, Villanueva, Li, et al., 2023)	• US probe (22 mm tip Φ); Freq.: 24 kHz; Power: 180 W; Time: 10 min; Cycle: 80 %; Temp.: 20, 40, 45, 50, 55 °C; Conc.: 25 % (w/w); Sample: 400 g<br>• SEM; XRD; HPSEC; FTIR; ^{1}H NMR; DSC	The effect of US depended on tef variety, where white tef presented higher susceptibility. Cavitation led to molecular depolymerization with preferential damage to the amorphous regions of the starch. The narrowing of the gelatinization enthalpy range indicated that US led to a more homogeneous starch configuration.
White tef flour<br>Brown tef flour<br><br>(Vela, Villanueva, Ozturk, et al., 2023)	• US probe (22 mm tip Φ); Freq.: 24 kHz; Power: 180 W; Time: 10 min; Cycle: 80 %; Temp.: 20, 40, 45, 50, 55 °C; Conc.: 25 % (w/w); Sample: 400 g<br>• PSD; Color; Starch damage; AAC; CLSM; Hydration properties; Pasting properties; Rheological properties	US led to particle fragmentation, which markedly increased starch damage and values of lightness (L^*). Amylose content was higher after ultrasonication, due to molecular fragmentation. Pasting properties showed increased pasting temperatures as well as decreased viscometric profiles with lower breakdown viscosities.
Flours from rice, tef, corn and quinoa<br>(Vela, Villanueva, Náthia-Neves, et al., 2023)	• US probe (22 mm tip Φ); Freq.: 24 kHz; Power: 180 W; Time: 10 min; Cycle: 80 %; Temp.: 20 °C; Conc.: 25 % (w/w); Sample: 400 g<br>• PSD; Color, Amylose content; Starch damage; FTIR; Techno-functional properties; DSC; Pasting properties; Rheological properties	The elimination of water by centrifugation resulted in the loss of solubilized compounds from the treated flours, which led to important differences between the final characteristics of US-treated flours. Gels made with tef, corn and quinoa presented reduced $tan(\delta)_1$ values after sonication, while gels made with rice did not show any modification.
Canary seed flour<br>(Náthia-Neves, et al., 2024)	• US probe (22 mm tip Φ); Freq.: 24 kHz; Power: 180 W; Time: 40 min; Cycle: 80 %; Temp.: 20, 30, 40 °C; Ratio: 1:7 (flour:water, w/w); Sample: 345 g	US treatment increased water and oil absorption capacities in canary seed flour. US treatment altered pasting and rheological properties of all studied flours, impacting viscosity and starch-lipid complex formation. US

	• WAC; OAC; WSI; SP; foaming capacity; emulsifying activity and stability; RVA; Rheological properties; DSC; XRD	treatment decreased gelatinization enthalpy in whole canary seed flour (up to -9.5%) and increased it in defatted (up to +11.3%) flours.
**GRAINS**		
Rice<br><br>(Cui et al., 2010)	• US bath; Freq.: 16 kHz; Power: 2000 W; Time: 30 min; Temp.: 25, 40 and 55 °C; Sample: 500 g<br>• Optimal cooking time; Volume expansion ratio; Water uptake ratio; Solid loss; SEM; Chemical composition and AAC; RVA; DSC; XRD	US resulted in loss in natural morphology of rice bran, allowing water to be absorbed by a rice kernel easily, particularly at high temperature treatment. Crystallinity was increased after treatment at 55 °C. US increased peak, hold, and final viscosities and decreased T_O. US treatment could be used for reducing cooking time of brown rice.
Rice<br><br>(Park & Han, 2016)	• US bath; Freq.: 400 kHz; Power: 185 W; Time, Temp.: 30 min at 25 °C, and 60 min at 50 °C; Conc.: 15 % (w/v); Sample: 300 g<br>• Textural properties of cooked brown rice; Determination of the thiamin, riboflavin and niacin contents; Water binding capacity and solubility of isolated starch; SEM; RVA; DSC; XRD	After US, the cooked brown rice grains were softer in proportion to soaking time. Thiamin and niacin contents in US treated brown rice were still higher than those in milled rice. Starch from brown rice grains treated at harsh condition exhibited a lower pasting temperature and higher breakdown than that treated at mild condition.
Buckwheat<br><br>(Harasym et al., 2020)	• US bath; Freq.: 45 kHz; Power: 100 W; Time: 15 min; 1:10, 1:5 and 1:2.5 solid:water ratio; Sample: 250, 500 and 1000 g<br>• PSD; Water absorption index (WAI); WSI; SP; RVA; Color; Soluble, insoluble, and total polyphenols content; Antioxidant activity	US caused specific agglomeration, resulting in bigger particles for 1:5 and 1:2.5 ratio treated samples, while higher dilution (1:10) increased smaller particle size fractions. Treatment increased flours' lightness and water solubility index. Soluble polyphenols content decreased, and insoluble polyphenols content increased after US treatment.
Indica rice<br>Japonica rice<br><br>(Shah et al., 2023)	• US probe; Power: 1200 W; Amp.: 30, 60 and 100 %; Time: 15, 30 and 60 min; Temp.: 20 – 25 ºC; Sample: 500g; Vol.: 1.5 L<br>• SEM; Protein and apparent amylose content; FTIR; XRD; DSC; RVA; Resistant starch; *In vitro* glycemic index (GI); Texture profile analysis	Starch granules merged and lost their shape when US time and amplitudes were increased up to 15 min and 30 %, respectively. US increased the crystallinity, gelatinization temperatures and decreased pasting viscosity, promoting more resistant starch and lower GI.

Freq. = Frequency. Amp. = Amplitude. Int. = Intensity. Temp. = Temperature. Atm. = Atmosphere. Solv. = Solvent. Conc. = Concentration. Vol. = Volume. US = Ultrasound. ANN = Annealing. HMT = Heat moisture treatment. MW = Microwave. RS = Resistant starch. SDS = Slowly digestible starch. RDS = Rapidly digestible starch.
